# Discovery at the Interface between Known Structure Types: The Layered Y_10_Ni_*x*_Cu_1–*x*_Ti_4_O_24_ Family

**DOI:** 10.1021/acs.inorgchem.6c02278

**Published:** 2026-07-12

**Authors:** Nataliya L. Gulay, Hai Lin, Batoul Almoussawi, Cara J. Hawkins, Manel Sonni, Marco Zanella, Troy D. Manning, Luke M. Daniels, Matthew S. Dyer, John B. Claridge, Matthew J. Rosseinsky

**Affiliations:** † Department of Chemistry, 4591 University of Liverpool, Materials Innovation Factory, 51 Oxford Street, Liverpool L7 3NY, U.K.; ‡ Leverhulme Research Centre for Functional Materials Design, Materials Innovation Factory, 51 Oxford Street, University of Liverpool, Liverpool L7 3NY, U.K.

## Abstract

During the exploration of the interface between the known structures of perovskite Y_2_NiTiO_6_ and hexagonal layered Y_2_CuTiO_6_, we have discovered the new phase Y_10_Ni_*x*_Cu_1–*x*_Ti_4_O_24_ (*x* = 0, 0.5, 1). The structure of Y_10_CuTi_4_O_24_ was solved by means of single-crystal X-ray diffraction, which revealed a layered monoclinic structure, with the space group *C*2/*m*, *a* = 12.2405(1), *b* = 5.8643(1), *c* = 7.1729(1) Å, β = 107.083(1)°. The structures of three Y_10_Ni_*x*_Cu_1–*x*_Ti_4_O_24_ (*x* = 0, 0.5, 1) phases were also refined based on high-resolution powder X-ray diffraction data. Substitution of Cu for Ni causes only minor changes in lattice and atomic parameters. The new phase is related to known Y_5_Mo_2_O_12_-type structures with an extra atomic position occupied by Ni/Cu in the structure of Y_10_Ni_*x*_Cu_1–*x*_Ti_4_O_24_ (*x* = 0, 0.5, 1). The high-resolution powder X-ray diffraction data revealed peak broadening for the reflections with *l* = 2*n* + 1 corresponding to stacking faults originating from the layered structure of Y_10_Ni_*x*_Cu_1–*x*_Ti_4_O_24_. Y_10_Ni_*x*_Cu_1–*x*_Ti_4_O_24_ (*x* = 0, 0.5, 1) were characterized with respect to their magnetic and optical properties.

## Graphical abstract

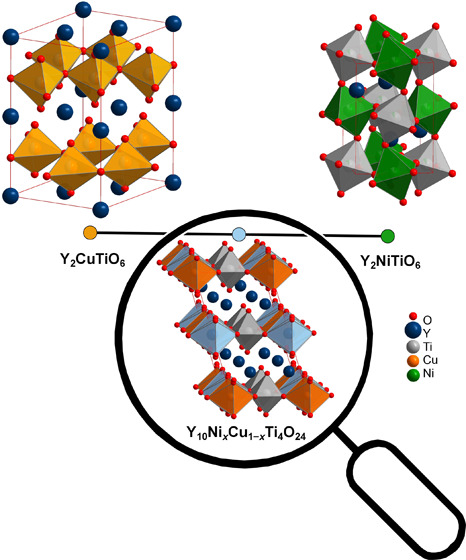

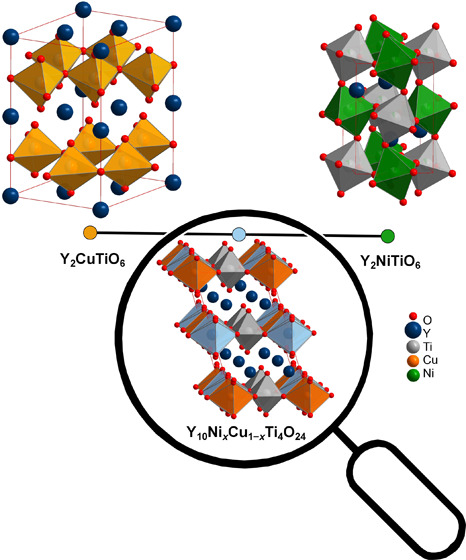

## 1 Introduction

Discovering new structures in an efficient way remains a challenging and critical task, given the foundational connection to the arising properties.^1^ With the recent focus on digital approaches,^2^ the pre-eminent role of chemical design based on understanding as a valid tool in materials design and chemical space exploration has become clearer.^3^ The hypothesis that new structure types can be found at the interface between known ones has already been demonstrated by the successful discovery of the two silicates Ba_5_Y_13_[SiO_4_]_8_O_8.5_ and Ba_3_Y_2_[Si_2_O_7_]_2_ in a particularly well-explored chemical space.^4^ Therefore, we aim to build on this hypothesis by exploring other phase boundaries.

Perovskites are a very common structural class of materials with thousands of members discovered over >185 years of research.^5^ The recent discovery of the double perovskite Y_2_NiTiO_6_ ^6^ motivated us to explore phase boundaries spanning from it to chemically similar but structurally different phases. A logical target was the interface spanning to Y_2_CuTiO_6_, which, despite similar composition, adopts the hexagonal structure of LuMnO_3_.^7^ There are no reported Y–Ni–Cu–Ti–O phases in the structural databases ICSD^8^ and Pearson’s.^9^

The end member Y_2_NiTiO_6_ exhibits an interesting connection between structural *B*-site order and magnetic properties,^6^ while Y_2_CuTiO_6_ attracted attention as a candidate green pigment.^10^ Further, Y–*M*–Ti–oxides (*M* = transitional metals) have been widely studied due to their diverse properties. For example, YAgTiO_4_ was studied with respect to negative thermal expansion,^11^ and Y_2/3_Cu_3_Ti_4_O_12_ was reported as a negative-temperature-coefficient ceramic.^12^ The solid solutions Y_2_(Zr_*y*_Ti_1–*y*_)_2_O_7_,^13^ Y_2_Ti_2_O_7_–Y_3_NbO_7_,^14^ and ZrO_2_–TiO_2_–Y_2_O_3_ ^15^ attracted interest with respect to their ionic conductivity. Y_2_Mo_2_(_1–*x*_)­Ti_2*x*_O_7_ features coexistence of antiferromagnetic and ferromagnetic interactions,^16^ while Y_2_MnTiO_7_ shows ferromagnetic behavior.^17^ YTiTaO_6_ attracted interest with respect to microwave dielectric properties.^18^ Finally, the broad YIn_1–*x*–2*y*–*z*_Mn_*x*_Ti_*y*_Zn_*y*_Al_*z*_O_3_ (*x* = 0.005–0.2, *y* = 0.1–0.4, and *z* ≤ 0.1) system with the hexagonal structure of LuMnO_3_ was studied with respect to tuning its color for stable nontoxic pigments.^19^

Exploring the interface between the recently discovered perovskite Y_2_NiTiO_6_^6^ and the hexagonal layered Y_2_CuTiO_6_,^7^ we have found the new phase Y_10_Ni_*x*_Cu_1–*x*_Ti_4_O_24_ (*x* = 0, 0.5, 1) with a monoclinic structure. These phases have a layered structure related to the far less-explored Y_5_Mo_2_O_12_ ^20^-type compounds, but with an extra atomic site occupied by the first transition series metal. The new phase contains 3.8 atom % of Ni and/or Cu and exists in proximity to another interface between binary Y_2_O_3_ and TiO_2_ (Figure 1). Synthesis, structural features, and magnetic and optical properties of these new phases are described herein.

**Figure 1:**
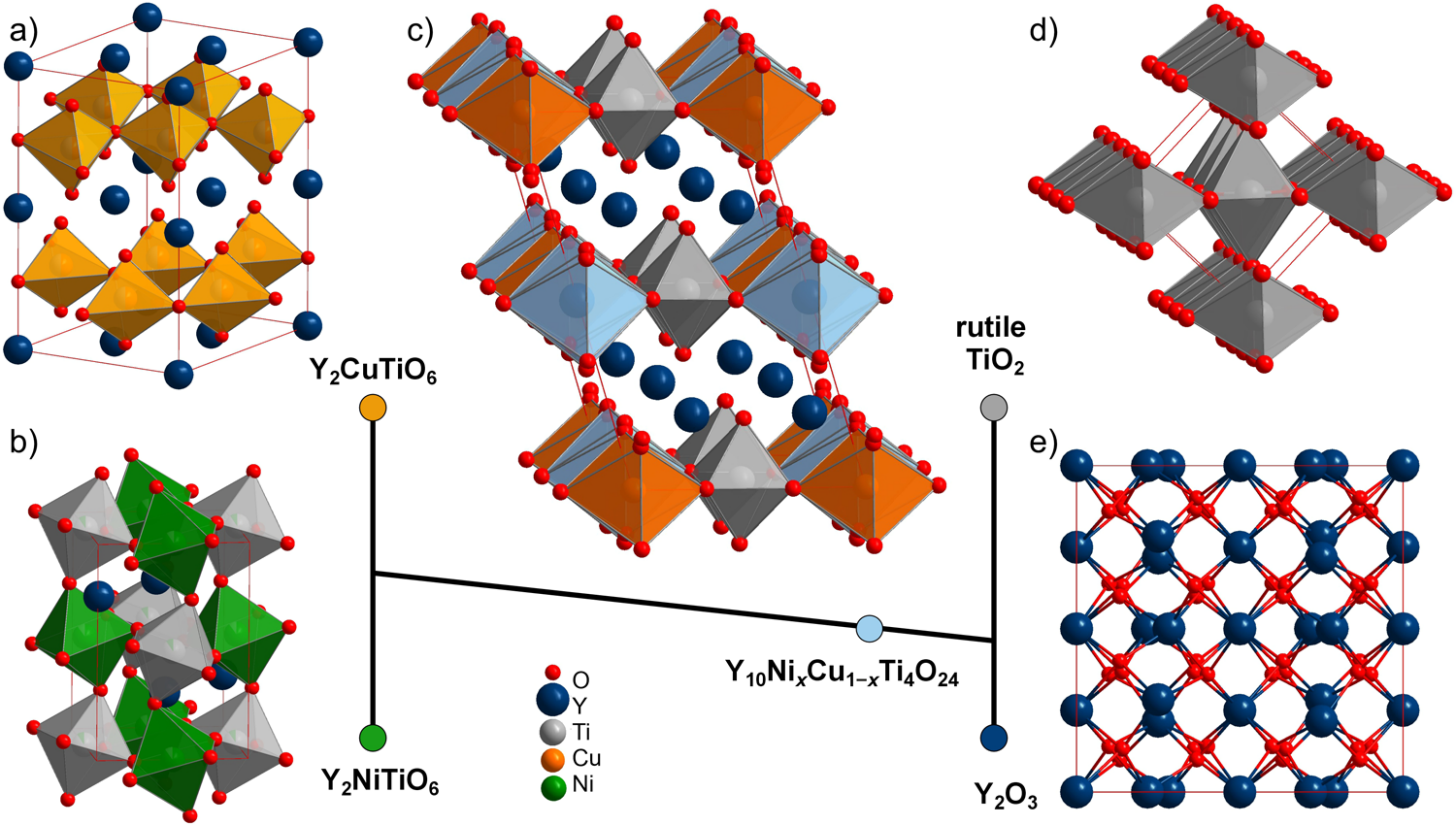
Schematic illustration of the interface between perovskite Y_2_NiTiO_6_^6^ (a) and hexagonal layered Y_2_CuTiO_6_^7^ (b), where the new phase Y_10_Ni_*x*_Cu_1–*x*_Ti_4_O_24_ (*x* = 0, 0.5, 1) (c) was discovered. The new phase contains only 1 Cu/Ni atom per 39 in the formula unit and lies much closer to the interface between binary Y_2_O_3_ (d) and TiO_2_ (e). The Y-, Ti-, Cu-,Ti/Cu-, and Ni-centered polyhedra are drawn in light blue, gray, orange, yellow, and green, respectively.

## 2 Experimental Part

### 2.1 Synthesis

Incorporation of as little as 3.8 atom % Ni and/or Cu causes the formation of the new phase Y_10_Ni_*x*_Cu_1–*x*_Ti_4_O_24_ (*x* = 0, 0.5, 1), which was not formed when either NiO or CuO was absent from the starting mixture, which instead resulted in a mixture of Y_2_O_3_, Y_2_Ti_2_O_7_, and Y_2_TiO_5_ (see Figure S1).

The bulk Y_10_Ni_*x*_Cu_1–*x*_Ti_4_O_24_ (*x* = 0, 0.5, 1) phases were formed using solid-state synthesis from oxide powders: yttrium­(III) oxide (Alfa Aesar, 99.999%), nickel­(II) oxide (Sigma-Aldrich, 99.99%), copper­(II) oxide (Alfa Aesar, 99.995%), and rutile titanium­(IV) oxide (Sigma-Aldrich, 99.8%). Before weighing, the starting powders were kept at 493 K in a furnace. The stoichiometric reactions were tested but produced significant amounts of competing Ni- and Cu-containing byproducts. Therefore, the starting ratio was optimized for each composition. For the best yield, the starting oxides were weighed to achieve the following optimized compositions: Y_10_Ni_0.89_Ti_3.72_O_23.33_ (for *x* = 0), Y_10_Cu_1.42_Ti_4_O_24_ (for *x* = 1), and Y_10_Cu_0.72_Ni_0.72_Ti_4_O_24_ (for *x* = 0.5). The powders were weighed to 0.5 g, homogenized using a mortar and pestle, placed in an alumina crucible, and prereacted at 1273 K for 12 h to minimize the potential losses of NiO and CuO. The heating and cooling rates were kept at 5 K/min unless stated otherwise. The prereacted powders were pressed in 10 mm pellets and used further to complete the synthesis.

All Y_10_Ni_*x*_Cu_1–*x*_Ti_4_O_24_ (*x* = 0, 0.5, 1) phases require different synthetic routines to navigate around competing byproducts. During the exploration, different crucible materials (alumina, zirconia, and zirconia covered with platinum foil), forms of the precursors (loose powders versus pellets), and temperature regimes were tested. Specific conditions described below correspond to those granting the best yield of the Y_10_Ni_*x*_Cu_1–*x*_Ti_4_O_24_ (*x* = 0, 0.5, 1) phases (81.66, 96.04, and 81.37 wt %, respectively) according to the results of PXRD analysis (see Table S3).

The Y_10_NiTi_4_O_24_ phase was obtained by placing the pellets of prereacted powders in zirconia crucibles and annealing them at 1673 K for 24 h. Afterward, the pellets were crushed, ground, and analyzed by powder X-ray analysis (PXRD). This step was repeated up to four times until no further change in PXRD patterns was observed. To obtain the Y_10_Ni_*x*_Cu_1–*x*_Ti_4_O_24_ (*x* = 0, 0.5) phases, the prereacted pelletized samples were annealed in zirconia crucibles at 1473 K for 12 h and at 1623 K for 24 h for Y_10_CuTi_4_O_24_ and Y_10_Ni_0.5_Cu_0.5_Ti_4_O_24_, respectively. The pellets were ground, and the powders were transferred in new zirconia crucibles to dwell for 5 days at 1473 K. This step was repeated 3–6 times until no change in PXRD patterns was observed. This step ensured that the dark-green Y_2_CuTiO_6_ byproduct was reacted to form the desired products, which could visually be verified by a color change. The final powders of Y_10_Ni_*x*_Cu_1–*x*_Ti_4_O_24_ (*x* = 0, 0.5, 1) phases have a light-yellow color and are stable in air for months.

Furthermore, it was possible to grow single crystals of Y_10_CuTi_4_O_24_ using a CuO flux. For this, the polycrystalline sample from the bulk synthesis was pressed into a pellet. The round platinum crucible (inner diameter ∼30 mm, height ∼25 mm) was filled with CuO powder (75 wt % of the pellet) and topped with the pellet piece (∼0.25 g) of the Y_10_CuTi_4_O_24_ sample. The platinum crucible was placed in a larger zirconia one (inner diameter ∼37 mm, height ∼32 mm, Almath crucibles CC35), closed with a lid, heated to 1573 K in a furnace with 5 K/min rate, and subsequently cooled to 1473 K with a 0.1 K/min rate. Afterward, the furnace was switched off, and the sample was cooled to room temperature via gradual heat loss. The resulting dark-green pellet was washed with small quantities (∼15 mL) of warm, diluted nitric acid to remove residual CuO. If a dark-green tint remained, this step was repeated, but caution should be taken, since the Y_10_Ni_*x*_Cu_1–*x*_Ti_4_O_24_ phases can be completely dissolved in acid. After washing, the yellow crystals grown on the pellet’s surface could be observed using an optical microscope. These crystals were mechanically removed from the bulk and used for single-crystal X-ray diffraction.

### 2.2 X-ray Diffraction

Routine examination of samples was conducted using in-house data collected on a PANalytical X’Pert diffractometer (monochromatic Co Kα_1_ radiation, λ = 1.78896 Å). The finely ground samples were uniformly distributed on greased glass slides and measured in reflection mode. Phase analysis was done by matching experimental diffraction patterns with patterns of reported phases using X’Pert HighScore Plus software.

High-resolution synchrotron diffraction data were collected at Diamond Light Source, beamline I11, using a Mythen position-sensitive detector (PSD) and a Multianalyzer crystal detector (MAC). The polycrystalline samples were sealed in 0.3 mm borosilicate capillaries and measured at room temperature (293 K). Powder X-ray diffraction data was handled using Topas Academic V7.^21^

Yellow single crystals from the Cu-containing sample were selected under an optical microscope and studied at Diamond Light Source, beamline I19 (λ = 0.6889 Å, Pilatus 2 M detector). The data were collected at 100 K. Cell refinement and data reduction were performed using Xia^22^ and Dials^23^ programs. The structures of these compounds were solved using Jana2020 software.^24^ Crystal structures were visualized using Diamond software (version 5.1.0).^25^

### 2.3 Energy Dispersive X-ray Spectroscopy (EDX)

The compositions of the samples were determined using energy dispersive X-ray spectroscopy (EDX) on a JEOL2100+ operating at 200 kV equipped with an SDD detector from Oxford Instruments (Model: X-Max 65T with a 65 mm^2^ surface area detection). For this, a small amount of the well-ground sample was dispersed in 4 mL of ethanol, and the resulting suspension was left decanting for 10–20 min until larger particles precipitated. An aliquot of the supernatant was deposited on a lacey carbon film-coated transmission electron microscopy (TEM) grid and allowed to dry in air. Afterward, the grids were placed on a TEM holder, which was tilted by 10° toward the detector to reduce the signal coming from the holder. Data were measured and analyzed using Aztec software. Correction factors were determined by measuring corresponding standards for each chemical element.

### 2.4 Measurements of Magnetic Properties

The Y_10_Ni_*x*_Cu_1–*x*_Ti_4_O_24_ (*x* = 0, 0.5, 1) samples ground to fine powders were weighed to ∼15 mg and placed in polypropylene capsules, which in turn were secured in a plastic straw holder. The holder was attached to a sample holder rod, which was inserted in a chamber of a commercial superconducting quantum interference device (SQUID) magnetometer from Quantum Design. Magnetic susceptibility data of the samples was recorded in a range of 2–300 K with an applied external magnetic field of 1 kOe in the field-cooled (FC) and zero-field-cooled (ZFC) modes.

Fitting of the data was done by using OriginPro 2024 b software.

### 2.5 Diffuse Reflectance Measurements

Diffuse reflectance spectra were measured between 200 and 2500 nm with a step size of 1 nm on an Agilent Cary 5000 instrument. Prior to the measurement, a poly­(tetrafluoroethylene) (PTFE) standard and a light trap were used for calibration to 100 and 0%, respectively. The band gap was determined using the Tauc plot method, as described by Makuła et al.^26^

## 3 Results and Discussion

### 3.1 Structure Refinement and Description from Single-Crystal Diffraction Data

During the exploration of the miscibility of Y_2_NiTiO_6_^6^ and Y_2_CuTiO_6_^7^ phases, the diffraction pattern of a sample with a nominal composition of Y_2_Ni_0.64_Cu_0.36_TiO_6_ showed a set of reflections that could not be assigned to known phases in the Y–Ni–Cu–Ti–O phase field. EDX analysis of that sample indicated a cluster of measured compositions centered around Y_66(2)_Ni_5(1)_Cu_7(2)_Ti_23(1)_O_158_, away from known compositions. A sample prepared at the Y_66_Ni_5_Cu_7_Ti_23_O_158_ stoichiometry using binary oxides as starting materials produced a considerable yield of the new phase. Based on I11 powder diffraction data, the reflections associated with this new phase were first indexed by a C-centered monoclinic unit cell (*a* = 21.424, *b* = 5.887, *c* = 7.168 Å, β = 146.79̊). The phase exists in a complete Ni–Cu compositional range, and it was possible to isolate both end-member compounds, Y_10_NiTi_4_O_24_ and Y_10_CuTi_4_O_24_.

Successful single-crystal growth experiments using CuO as a flux enabled the solution of the crystal structure of Y_10_CuTi_4_O_24_. The *C*2/*m* symmetry was confirmed with lattice parameters *a* = 12.2405(1), *b* = 5.8643(1), *c* = 7.1729(1) Å, and β = 107.083(1)° that correspond to a standard setting of those obtained from PXRD. The charge-flipping algorithm embedded in SuperFlip^27^ within Jana2020 ^24^ was used to obtain the starting model with 4 O, 3 Y, 1 Ti, and 1 Cu sites. The structure was refined by least-squares against *F* ^2^ using Jana2020 software. The occupancies of all sites were refined independently to identify possible defects. Only the 2*a* Cu site was refined to be 0.509(4), while all other sites were assigned full occupancies within 3 times the standard deviation. The refined composition of Y_10_Cu_1.018(8)_Ti_4_O_24_ is charge-balanced within three standard deviations and correlates well with the EDX data (Y_10.0(4)_Cu_1.7(2)_Ti_4.1(3)_, Figure S2). Refinement of the anisotropic displacement parameters revealed large anisotropy for the 2*b* Y3 site along the *b*-axis; therefore, it was displaced from the ideal position and refined as a 4*g* position with occupancy set to 50%. The 8*j* O1 site also showed large anisotropic displacements; therefore, it was refined as two 50% occupied 8*j* positions (O1a and O1b). The displacement parameters for these split sites were constrained to the same values and were refined isotropically. Splitting these sites does not affect the refinement statistics but provides a better model for the disorder in this structure corresponding to the environment of 0.5 occupied Cu sites, which will be discussed in more detail below.

The final model of Y_10_CuTi_4_O_24_ was refined with no significant residual electron density in the difference Fourier map. The structure refinement details for Y_10_CuTi_4_O_24_ based on the single-crystal data are summarized in Table 1, while atomic coordinates, displacement parameters, and interatomic distances are listed in Tables S1 and S2.

**Table 1:** Crystallographic Data and Structure Refinement Details for Y_10_CuTi_4_O_24_ (Space Group *C*2/*m*, *Z* = 1) from I19 (λ = 0.6889 Å) Single Diffraction Data Collected at 100 K

refined formula	Y_10_Cu1.018(8)Ti_4_O_24_
molar mass, g mol^–1^	1529.3
lattice parameters, Å	*a* = 12.2405(1),
	*b* = 5.8643(1),
	*c* = 7.1729(1),
	β = 107.083(1)°
cell volume, Å^3^	492.168(12)
calculated density, g cm^–3^	5.16
detector distance, mm	160
exposure time, s	0.2
ω-range/step width,°	–145° – +25°
absorption coefficient, mm^–1^	29.6
*F*(000), e	700
θ range,°	2.88–36.02
hkl range	–20, 20; ± 9, 9; −11, 12
total no. reflections	5567
independent reflections/*R*_int_	1291/0.1055
refl. with *I* ≥ 3σ(*I*)/*R*_σ_	1013/0.0522
data/parameters	1291/57
goodness-of-fit on *F* ^2^	1.44
*R*1/*w*R** 2 for *I* ≥ 3σ(*I*)	0.0419/0.0900
*R*1/*w*R** 2 for all data	0.0459/0.0915
extinction coefficient	300(50)
largest diff. peak/hole, e Å^–3^	1.37/–1.05

Y_10_CuTi_4_O_24_ crystallizes in the space group *C*2/*m*, Pearson code *mS*39, and Wyckoff sequence j^3^i^4^hga. The structure of Y_10_CuTi_4_O_24_ is shown in Figure 2. It can be described as the stacking of two types of layers along the *a*-axis, which are separated by a dashed line in Figure 2a,b. One layer (denoted as the “yttria layer” in Figure 2c) is composed of 7-coordinated Y1@O_7_ and Y2@O_7_ polyhedra interconnected via common edges. Within this layer, the Y–O distances range from 2.252 to 2.373 Å, which correlate well with the typical bond length of, e.g., Y_2_O_3_ (2.247–2.345 Å).^28^ The coordination of yttrium and the arrangement of the polyhedra in this layer closely resemble the fragments of the 3-dimensional network built of Y@O_7_ in the structure of BaCuY_2_O_5_ (Figure 2e).^29^

**Figure 2:**
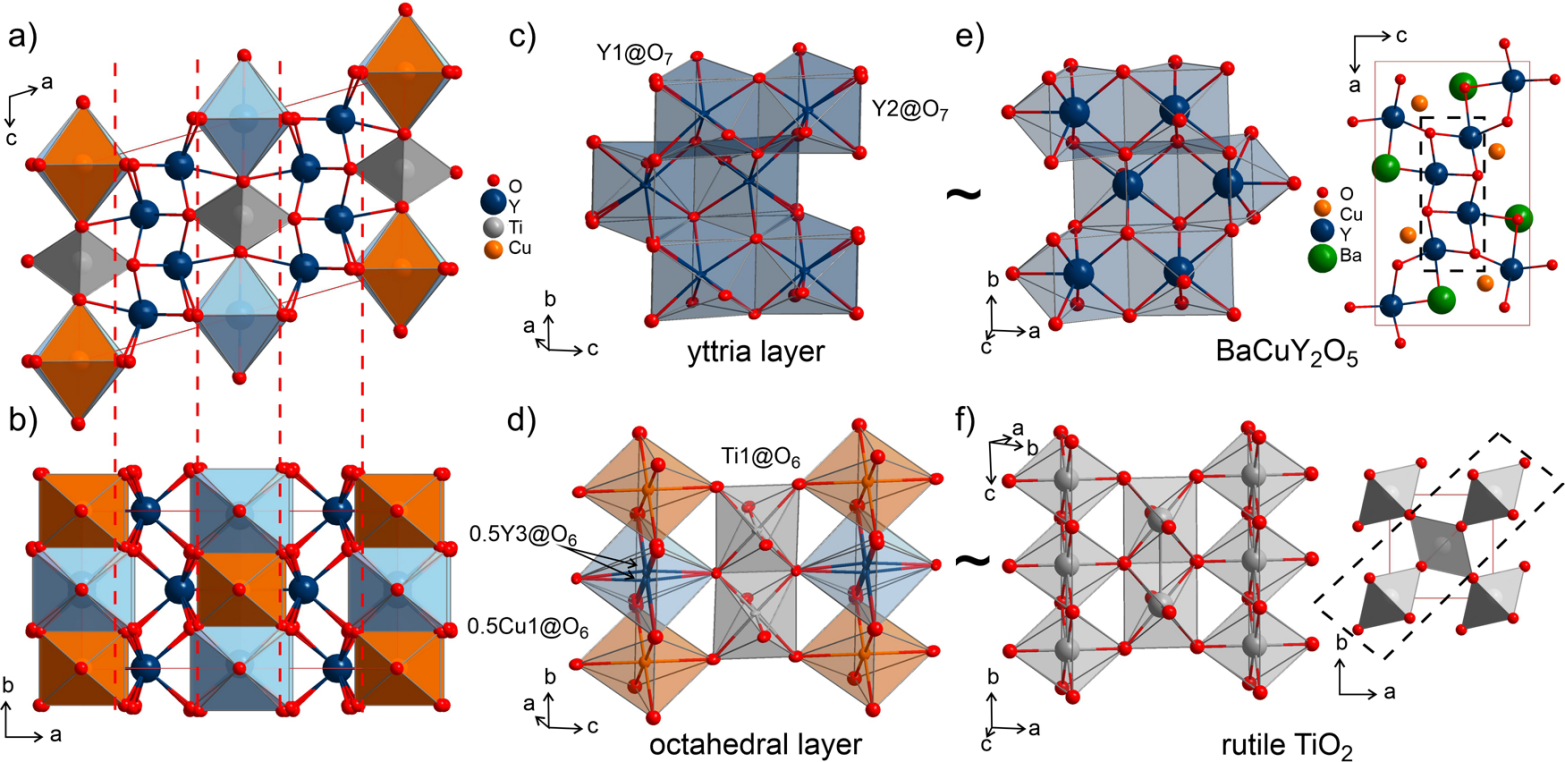
Structure of Y_10_CuTi_4_O_24_ drawn as *ac* (a) and *ab* (b) projections with dashed lines marking two layers composing this structure. (c) The yttria layer is composed of interconnected Y1@O_7_ and Y2@O_7_ polyhedra drawn in dark blue. (d) The octahedral layer consists of Ti-, 0.5Cu-, and Y3-centered octahedra drawn in gray, orange, and light blue, respectively. The yttria layer resembles (e) a fragment of the three-dimensional yttria network in the structure of BaCuY_2_O_5_,^29^ while the octahedral layer follows the arrangement of the octahedra in rutile TiO_2_ (f).^30^

The yttria layers connect via common faces and edges with the layers of Ti-, 0.5Cu-, and Y3-octahedra (“octahedral layer” in Figure 2d). These octahedra are arranged in the same manner as Ti@O_6_ within the diagonal slice across the *ab* plane in the structure of rutile TiO_2_ (Figure 2f).^30^ The octahedral layer consists of two columns (A and B in Figure 3a) of edge-sharing octahedra aligned along the *b*-axis. Column A (Figure 3b) consists of slightly distorted Ti@O_6_ octahedra with Ti–O distances from 1.915 to 2.037 Å, which are similar to the analogous distances in pyrochlore Y_2_Ti_2_O_7_ (Ti–O 1.925 Å)^31^ or perovskite Y_2_NiTiO_6_ (Ti–O 1.956–1.987 Å).^6^ The characteristic feature of this column is alternating short and long Ti–Ti distances of 2.814 and 3.049 Å, respectively. These values are averaged close to Ti–Ti distances in the structure of rutile TiO_2_ (2.959 Å).^32^ They are also quite similar to those observed in Ti-rich intermetallic compounds such as Ti_5_Mn_0.45_Sb_2.55_ (Ti–Ti of 2.639–3.059 Å)^33^ or Ti_4.8_Sb_3.3_ (Ti–Ti of 2.757–3.247 Å).^34^

**Figure 3:**
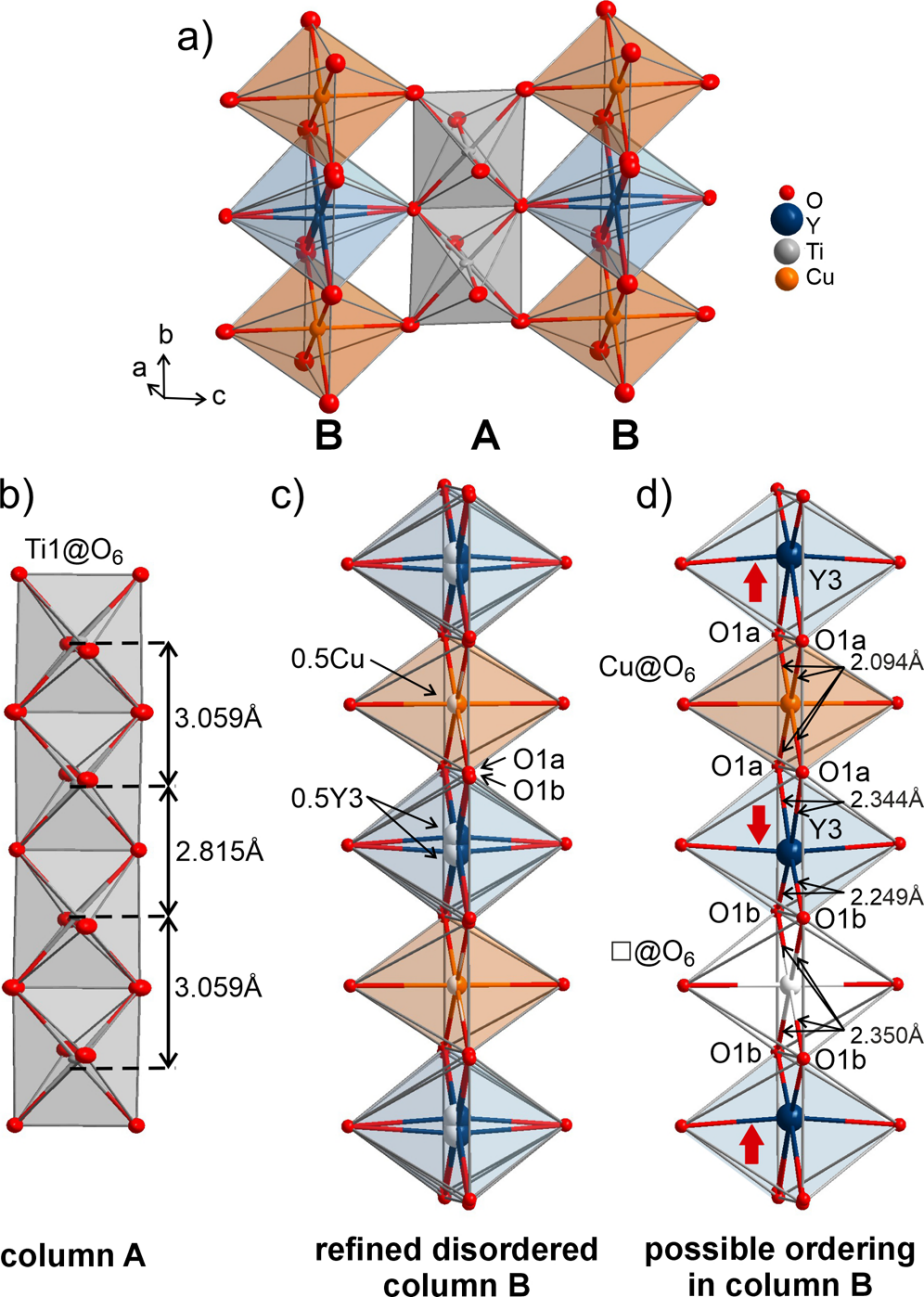
(a) Octahedral layer in the structure of Y_10_CuTi_4_O_24_ consisting of two types of columns. (b) Column A, which consists of edge-sharing Ti-centered octahedra and features an alteration of shorter and longer Ti–Ti distances. (c) Column B of alternating edge-sharing Y3- and 0.5Cu-centered octahedra connected via split O1a/O1b sites showing the refined statistical disorder in the structure. (d) Possible physical realization of the order within column B showing the alteration of full Cu@O_6_ (4 × O1a and 2 × O3) and empty □@O_6_ (4 × O1b and 2 × O3) octahedra and shift of Y along the *b*-axis toward the empty octahedral space created by the Cu vacancy. The relevant Y–O and Cu–O bond lengths are emphasized. The yttrium-, titanium-, and copper-centered octahedra are drawn in light blue, gray, and orange, respectively.

Column B (Figure 3c) consists of octahedra alternately filled with Y3 (split as two 50% occupied sites as described above) and 50% occupied Cu atoms, which are connected via the edge split O1a/O1b atoms. With respect to site occupancies, the total coordination of both Y3 and Cu sites sums up to six. Interestingly, the larger Y3@O_6_ (Y–O of 2.112–2.454 Å) are positioned next to the shorter Ti–Ti distance, while the smaller 0.5Cu@O_6_ (Cu–O of 2.094–2.350 Å) are opposite the longer one.

Disorder represented by the splitting of Y3 and O1a/O1b atoms within column B is a direct consequence of the statistical 50% occupancy of the Cu site. This site represents a superposition of octahedra that are 100% filled by Cu and are empty (Figure 3d). When occupied, the Cu site is surrounded by four O1a at 2.094 Å and two O3 at 2.236 Å atoms comprising a Q3 Jahn–Teller elongated octahedron.^35^ When empty, the □@O_6_ octahedron consists of 4 O1b at 2.350 Å and 2 O3 at 2.236 Å atoms from the 2*a* site. Consequently, the Cu/□ disorder forces a shift of the Y3 atom from its ideal 0, 1/2, 0 position toward the empty octahedra. The coordination of full Cu@O_6_ (4 × O1a and 2 × O3) shows similar distortions to those observed in the structures of Cu-containing double perovskites, e.g., Sr_2_CuWO_6_, which features four 1.955 and two 2.322 Å Cu–O bonds.^36^ In the structure of Y_10_CuTi_4_O_24_, the shorter Cu–O bond length is slightly longer than for the typical square-planar coordinated Cu­(II) compounds such as CaCu_3_Ti_4_O_12_ (Cu–O of 1.961 Å)^37^ or CuO (1.951–1.961 Å).^38^ The Y3–O interatomic distances lie in the broad range from 2.112 to 2.454 Å, which corresponds to a superposition of two Y-centered octahedra with much more reasonable 2.249–2.344 Å bond lengths similar to the analogous sites in the structure of Y_5_Mo_2_O_12_ (2.223–2.302 Å).^20^

We have attempted to model the potential ordering of the Cu1/□@O_6_ octahedra with different supercells. However, since no extra peaks consistent with these superstructures were observed on high-resolution synchrotron data for either single crystals or powders, we can assume that the average structure is statistically disordered, representing the range of possible short-range orderings with no single motif preferred.

Finally, we can relate Y_10_CuTi_4_O_24_ (a new structure type with a unique Wyckoff sequence, and a site coloring not matched with reported structures in Pearson’s^9^ or ICSD^8^ databases) with the known Y_5_Mo_2_O_12_ ^20^ structure. As evident from Figure 4a,b, both structures share a common layered motif with yttria layers being identical in Y_5_Mo_2_O_12_ and Y_10_CuTi_4_O_24_. However, the crucial differences arise within the octahedral layers of these structures. As highlighted in Figure 4c, Y_5_Mo_2_O_12_ has a completely vacant octahedral space in between Y3@O_6_ octahedra. In contrast, the structure of Y_10_CuTi_4_O_24_ features a 50% occupied Cu site in this position (Figure 4d), creating a connectivity present in rutile. The existence of this site stabilizes this structure, making it charge-balanced as well as diversifying the coloring (distribution of the elements)^39^ on the sites of the octahedral layer.

**Figure 4:**
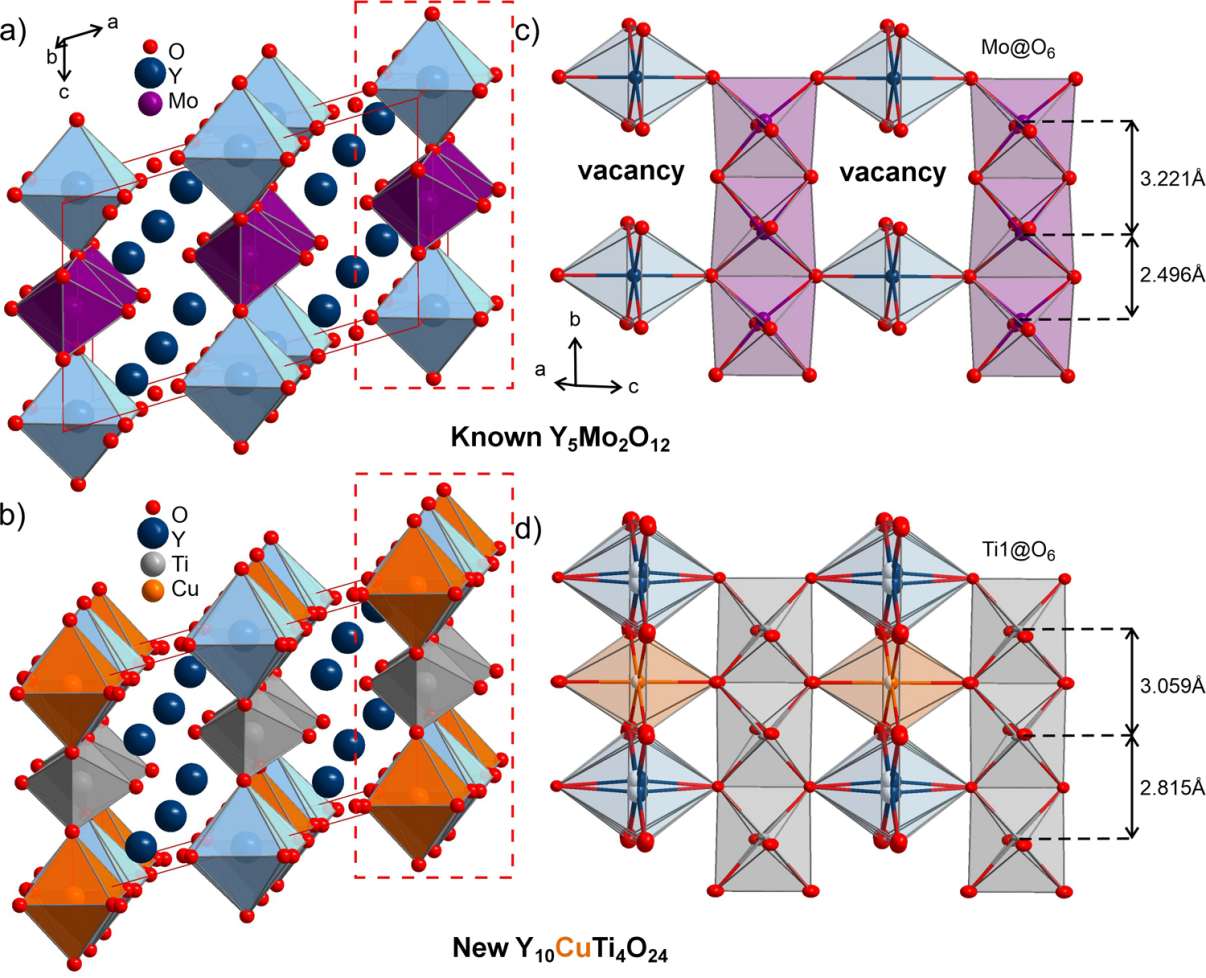
Structures of known Y_5_Mo_2_O_12_ ^20^ (top) and new Y_10_CuTi_4_O_24_ (bottom). (a, b) Projections of the unit cells onto the *ac* plane, showing a similar layered arrangement. The dashed line emphasizes the octahedral layers (A/B in Figure 2), where differences arise. These layers are enlarged in (c, d) to show the presence of octahedral vacant sites in Y_5_Mo_2_O_12_ (c), which are occupied by 0.5Cu in the structure of Y_10_CuTi_4_O_24_. Both structures feature alteration of short and long Ti–Ti and Mo–Mo distances; however, those distances differ to a greater extent in Y_5_Mo_2_O_12_ than in Y_10_CuTi_4_O_24_. The titanium-, molybdenum-, and copper-centered octahedra are drawn in gray, violet, and orange, respectively.

Structures of both Y_10_CuTi_4_O_24_ and Y_5_Mo_2_O_12_ feature the alteration of short and long Ti–Ti and Mo–Mo distances, respectively (Figure 4c,d). In the structure of the Y_10_CuTi_4_O_24_ phase, the difference between the shortest and longest Ti–Ti distances is still distinct, but it is less drastic than for the Mo,^20,40,41^ Ru,^42,43^ and Re^44–46^ members of the Y_5_Mo_2_O_12_ type. In the case of the latter structures, this was explained by the +4/+5 mixed valency of the transitional metal atoms, which is not applicable to the open shell Ti^4+^.

### 3.2 Defects in Y_10_Ni_*x*_Cu_1–*x*_Ti_4_O_24_ (*x* = 0, 0.5, 1) Revealed from Powder Diffraction

The model obtained from single-crystal X-ray diffraction for Y_10_CuTi_4_O_24_ was used for the Rietveld refinement of the structures of Y_10_Ni_*x*_Cu_1–*x*_Ti_4_O_24_ (*x* = 0, 0.5, 1) members against high-resolution synchrotron powder diffraction data (Figure S4). The refined cell parameters for the Y_10_Ni_*x*_Cu_1–*x*_Ti_4_O_24_ (*x* = 0, 0.5, 1) phases are listed in Table 2, and they agree well with the single-crystal data for Y_10_CuTi_4_O_24_. However, direct fitting of the single-crystal model to the powder data revealed peculiarities in some of the peak shapes of the new phase. Upon examination, low intensity peaks with *l* = 2*n* exhibit a much broader shape than those with *l* = 2*n* + 1. These features were most prominent for Y_10_CuTi_4_O_24_ and Y_10_NiTi_4_O_24_ end members, while the mixed Y_10_Ni_0.5_Cu_0.5_Ti_4_O_24_ compound displayed the sharpest peaks (see Figures 5a and S3). Similar peculiarities in the peak shape of even and odd *l*-indices were observed for *RE*_5_Mo_2_O_12_ (*RE* = La, Y, Lu) and were associated with stacking faults.^41^ Therefore, we refined the single-crystal model with two distinct peak shape functions for the broad even and sharp odd *l*-values for the Rietveld refinement, using the methodology that Barton et al. first applied to describe the short-range order of LiNiO_2_–NiO.^47^ Utilization of two peak shape functions in the refinement yielded a significantly better fit to the experimental data and improvement of the refinement statistics for all of the Y_10_Ni_*x*_Cu_1–*x*_Ti_4_O_24_ (*x* = 0, 0.5, 1) phases, compared to models with only a single peak shape function (Figures 5b and S4). Furthermore, it allows the extraction of the two volume-weighted^48^ column height components (*L*) describing sharp and broad peaks, *L*_even_ and *L*_odd_. At the same time, small discrepancies in the fits of the *l*-odd peaks remained (see sharper (021), (311), (22–3), and (51–3) and broader (22–1), (221), and (42–1) peaks in Figure 5b). These features can be fitted using a model to account for possible stacking faults following a methodology applied for the Li_3_HoBr_6–*x*_I_*x*_ ^49^ and Ag_3_LiRu_2_O_6_ ^50^ phases and using the toolkit of the Bilbao Crystallographic Service for cell transformations^51^ (details are given in SI Sections S2–S4). The stacking was described as a probability (*p*) of the unit cell to translate into the equivalent cell, shifted by 1/2 along the *c*-axis. A completely faultless structure would have this stacking probability *p* of 0, while a value of 1 would indicate shifts after each translation. The refinement with *p* = 0.041 further improved the statistics and fit of the experimental data compared to the two-profile function and correctly described uniformity in intensities and peak shape (see Figure 5c,f).

**Table 2:** Unit Cell Dimensions for the Y_10_Ni_*x*_Cu_1–*x*_Ti_4_O_24_ (*x* = 0, 0.5, 1) Phases Refined against Synchrotron (λ = 0.824672 Å) PXRD Data Collected at Room Temperature (298 K)

phase	*a*, Å	*b*, Å	*c*, Å	β, °	volume, Å^3^
Y_10_NiTi_4_O_24_	12.24649(6)	5.87759(2)	7.14454(2)	106.9683(8)	491.876(4)
Y_10_Ni_0.5_Cu_0.5_Ti_4_O_24_	12.24609(3)	5.87615(1)	7.15521(2)	107.0169(2)	492.345(2)
Y_10_CuTi_4_O_24_	12.24513(3)	5.86987(1)	7.15164(2)	107.0645(3)	493.471(2)

**Figure 5:**
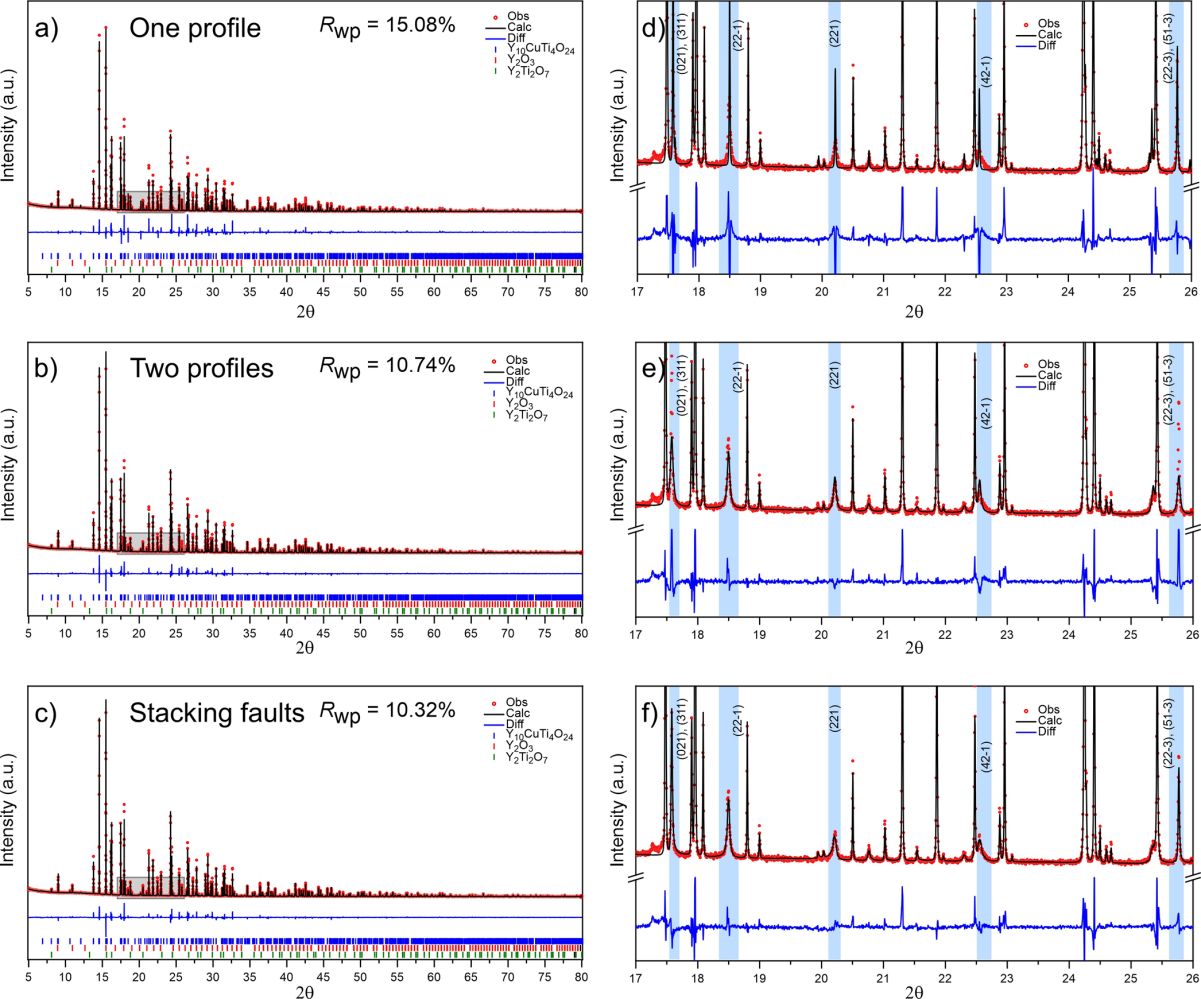
(Left) Rietveld refinement of the structure of Y_10_CuTi_4_O_24_ (space group *C*2/*m*, *Z* = 1) against synchrotron PXRD data (λ = 0.824672 Å) collected at room temperature (298 K) using (a) one versus (b) two peak shape functions, and (c) modeling stacking faults. (Right) (d–f) Enlarged regions from 17 to 26° 2θ (highlighted with gray rectangles in a–c) that feature non-uniform peak shapes for odd *l*-indices (light-blue shading). The most relevant *hkl* indices are noted. Observed, calculated, and the difference intensity are drawn in red circles, black line, and blue line, respectively.

The peak broadening observed in the powder patterns is connected to stacking fault defects caused by the layered structure of Y_10_Ni_*x*_Cu_1–*x*_Ti_4_O_24_ (*x* = 0, 0.5, 1). In the ideally ordered structure, the octahedral layers are stacked along the *a*-axis with B columns centered on the *ac* plane, while A columns are parallel to them but shifted along the *c*-axis for 1/2 of the cell length (Figure 6a,b). The stacking fault can occur when the octahedral layers shift for the 1/2 *c-*axis switching positions of A and B columns without disturbing the uniformity of yttria layers (Figure 6b,c). When this stacking fault happens, it breaks the translation periodicity along the *a*-axis, as now A and B columns can coexist within the same plane (Figure 6c,d). Therefore, the real stacked faulted structure features a superposition of A and B columns within a theoretical smaller cell with *c*_stacked_ = 1/2 *c*_ideal_ (emphasized by the red dotted line in Figure 6) corresponding to the sharp *l* = 2*n* peaks. With an increasing number of stacking faults, the ideally ordered fragments of the structure (blue lines in Figure 6) will have a smaller coherence length, which will cause broadening of the *l* = 2*n* peaks with respect to the sharp ones. The refined values of *L*_even_ and *L*_odd_ can give us a relative estimate of the correlation length ratio between crystallites with an ideal structure (*L*_even_) within real stacking faulted grains (*L*_odd_). For the samples with broader peaks (e.g., Y_10_NiTi_4_O_24_ in Figure 7(a), the *L*_even_: *L*_odd_ ratio is 17.4, reflecting a higher degree of stacking faults than that for the Y_10_Ni_0.5_Cu_0.5_Ti_4_O_24_ phase (Figure 7b) with the sharpest *l* = 2*n*+1 peaks and a lower *L*_even_:*L*_odd_ of 5.8. The refined values of *L* for all three Y_10_Ni_*x*_Cu_1–*x*_Ti_4_O_24_ (*x* = 0, 0.5, 1) samples are listed in Table S3.

**Figure 6:**
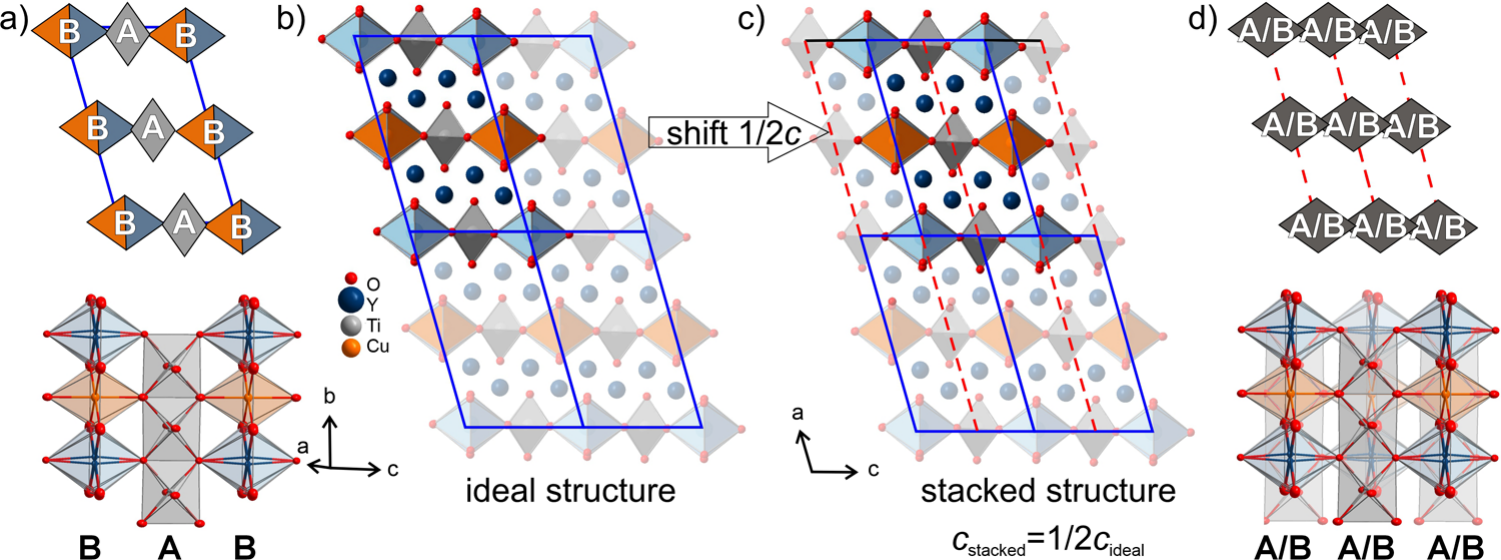
Stacking fault mechanism in the structure of Y_10_Ni_*x*_Cu_1–*x*_Ti_4_O_24_ (*x* = 0, 0.5, 1) phases. Columns A and B (a) within the octahedral layers in the ideal ordered structure (b) can shift along the *c*-axis for 1/2 of the cell length without disturbing the yttria network. The resulting stacked structure (c) can have switched A and B columns and can also be described with the smaller cell with *c*_stacked_ = 1/2 *c*_ideal_ featuring A/B superposition (d).

**Figure 7:**
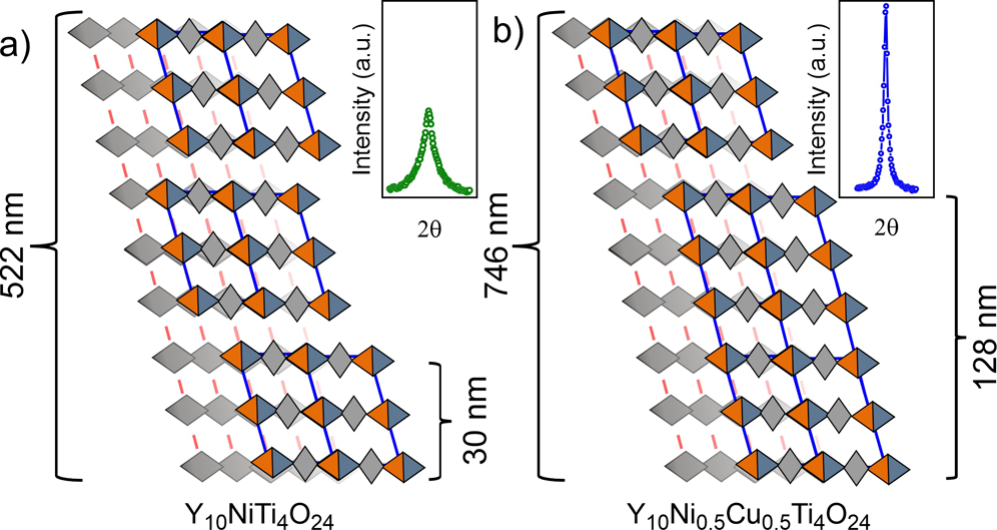
Schematic visualization of the stacking fault degree as approximated from the two volume-weighted^48^ column height components *L*_even_ and *L*_odd_ describing sharp and broad peaks, respectively, exemplified for Y_10_NiTi_4_O_24_ (a) and Y_10_Ni_0.5_Cu_0.5_Ti_4_O_24_ (b). The refined values of *L*_even_ and *L*_odd_ are shown next to bigger and smaller brackets, respectively. The insets show enlarged 22–1 reflection to illustrate different peak shapes in different samples (see Figure S3 for the extended patterns).

After accounting for two different peak shapes, the refined models from the XRD data correlate well with the one obtained from the single-crystal data for Y_10_CuTi_4_O_24_. Refined parameters and atomic coordinates are listed in Tables S3 and S4. The occupancies of all atomic sites were refined separately, but the deviations from unity remained within 3 times the standard deviation. Similarly to the single-crystal refinement, the total occupancy of the 2*a* Cu/Ni site was refined close to half-occupied and fixed at 0.5, which is consistent with the EDX data within 3 times the standard deviation (Y_10.0(4)_Cu_1.7(2)_Ti_4.1(3)_ for *x* = 0; Y_10.0(6)_Ni_0.7(1)_Cu_0.8(4)_Ti_3.6(1)_ for *x* = 0.5; Y_10(2)_Ni_0.82(5)_Ti_3.6(1)_ for *x* = 0, Figure S2) and charge-balanced formula. For Y_10_Ni_0.5_Cu_0.5_Ti_4_O_24_, the Ni:Cu ratio was fixed at 1:1, corresponding to a 0.25 occupancy of the split site by each metal. The substitution of Cu by Ni causes minor changes in the structure of the Y_10_Ni_*x*_Cu_1–*x*_Ti_4_O_24_ (*x* = 0, 0.5, 1) phase, with lattice parameters and atomic coordinates having very close values for three compositions (see Tables 1, S3, and S4). The biggest difference occurs in the coordination of the half-occupied Ni/Cu site (see Table S5). In the structure of Y_10_NiTi_4_O_24_, the four shortest Ni–O1a bonds expand to 2.192 Å, which is close to the next two Ni–O3 of 2.210 Å, making the Ni site coordination nearly perfectly octahedral. In contrast, both Cu and Cu/Ni sites in the respective structures feature four much shorter bonds to O1a atoms of 2.089 Å, corresponding to Jahn–Teller elongated octahedra in agreement with the model obtained from the single-crystal data. With Cu for Ni substitution, the shorter Ti–Ti distances shrink slightly from 2.821 to 2.815 Å, while the longer ones expand from 3.049 to 3.062 Å.

### 3.3 Magnetic Properties

All samples are characterized with Curie–Weiss paramagnetism, with no ordering observed down to 2 K. The temperature-dependent magnetic properties of three Y_10_Ni_*x*_Cu_1–*x*_Ti_4_O_24_ powder samples were measured on the SQUID-VSM Quantum Design at an external magnetic field of 10000 Oe (Figure 8a, *x* = 0, 0.5, 1, drawn in red, blue, and gray, respectively). The measurements performed with zero-field cooling (ZFC) and field cooling (FC) are denoted with open and solid circles, respectively. For all samples, neither significant magnetic transitions nor divergences between measurements performed in ZFC and FC conditions are observed during the measured temperature range from 2 to 300 K. Moreover, the inverse magnetic susceptibility χ^–1^ = *H*/*M* (Figure 8b) exhibits linear temperature dependence, clearly revealing Curie paramagnetism in this system. The presence of impurities in the polycrystalline samples (see Table S3) has a negligible effect on the magnetic trends, and even the Y_10_NiTi_4_O_24_ sample with 3.5 wt % Y_2_NiTiO_6_ does not exhibit any ordering features below 20 K characteristic of the latter.^6^

**Figure 8:**
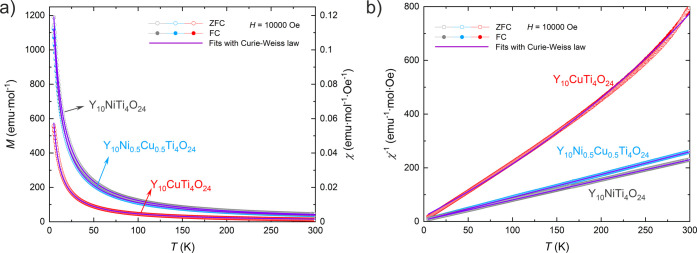
(a) Magnetizations *M* and (b) inverse magnetic susceptibilities χ^–1^ of Y_10_Ni_*x*_Cu_1–*x*_Ti_4_O_24_ samples (*x* = 0, 0.5, 1, drawn in red, blue, and gray, respectively), measured from 2 to 300 K at the external magnetic field of 10000 Oe. The open and solid circles denote the results measured with zero-field cooling (ZFC) and field cooling (FC) processes, respectively. No significant divergence observed between the two processes and the linear temperature dependence of χ^–1^ suggest the Curie paramagnetism in all three compounds. The fits with the Curie–Weiss law (violet lines) on the field-cooling data therefore agree well with the data.

The magnetic moments are therefore determined by using the extended Curie–Weiss law^52^ as follows, fitting the χ^–1^ data to avoid overweighting the low-temperature data

$$\chi^{-1}={\left(\frac{C}{T-T_{\mathrm{CW}}}+\chi_{0}\right)}^{-1}$$

Here, *C* is the Curie constant, *T*_CW_ is the Curie–Weiss temperature, and χ_0_ is the temperature-independent susceptibility term. The χ_0_ obtained from the fits (Table S7), 2 to 3 orders of magnitude smaller than χ, causes the very slight positive curvature in the χ^–1^ plot, which can come from any temperature-independent magnetism contributed by the sample, sample holder, or impurities below the detection limit of PXRD,^52^ e.g., residual NiO.^53^ The fits (violet lines in Figure 8) are in excellent agreement with the χ^–1^ data as well as the back-inverted χ. The effective magnetic moment in units of Bohr magnetons (μ_B_) can therefore be calculated from the Curie constant in cgs units

$$\mu_{\mathrm{eff}}=\sqrt{8C}\mu_{B}$$

which gives μ_eff_ = 2.148(3) μ_B_ for Y_10_CuTi_4_O_24_, 3.063(1) μ_B_ for Y_10_Ni_0.5_Cu_0.5_Ti_4_O_24_, and 3.251(1) μ_B_ for Y_10_NiTi_4_O_24_. It should be noted that all three compounds exhibit a larger effective moment than the calculated spin-only magnetic moment $\mu_{s}=g\sqrt{S\left(S+1\right)}\mu_{B}$ for valence-state ions in octahedral coordination (1.73 μ_B_ for Cu^2+^, 2.28 μ_B_ for 0.5Cu^2+^ + 0.5Ni^2+^, and 2.83 μ_B_ for Ni^2+^), where the *g* factor assumes the spin-only value of 2 and the spin angular momentum *S* equals 1 for Ni^2+^. The small deviation between μ_eff_ and μ_s_ results from the unaccounted orbital contribution in the spin-only formula. This is commonly observed in such compounds of which the *g* factor deviates from the spin-only value: *g* = 2 (1–4 λ/Δ), where λ is the spin–orbit coupling constant (commonly negative for nickelates) and Δ is the crystal-field splitting energy between the ground and excited states.^54^ The μ_eff_ in such compounds are reported as wider ranges of 1.7–2.2 μ_B_ for Cu^2+^ and 2.9–3.3 μ_B_ for Ni^2+^,^52^ which is commonly associated with local ionic environment and spin–orbit coupling.^52^ For reference, a μ_eff_ per Ni^2+^ ion of 3.28 μ_B_ was reported for PbCd_2_Ni_6_Te_3_O_18_ ^55^ while Y_2_NiTiO_6_ has effective magnetic moments of 2.85(1) and 2.95(2) μ_B_ for the orthorhombic and monoclinic modifications, respectively.^6^ A μ_eff_ of 2.0 μ_B_ was observed for Y_2_CuTiO_6_.^56^

### 3.4 Optical Properties

In contrast to the dark-green Y_2_NiTiO_6_^6^ and green Y_2_CuTiO_6_^10^, the Y_10_Ni_*x*_Cu_1–*x*_Ti_4_O_24_ (*x* = 0, 0.5, 1) phases have light-yellow colors, which can be explained by their much smaller Ni/Cu content. Figure 9a shows the absorbance spectra for all three phases. The three absorbance peaks at around ∼400, 700, and 1200 nm in Y_10_NiTi_4_O_24_ are also observed in Y_2_NiTiO_6_^6^ and are consistent with those characteristic for the Ni^2+^ compounds at 400, 800, and 1400 nm, corresponding to ^3^A_2g_ → ^3^T_1g_(F), ^3^A_2g_ → ^3^T_1g_(G), and ^3^A_2g_ → ^3^T_2g_ transitions, respectively.^57^ The absorbance spectrum of Y_10_CuTi_4_O_24_ features two distinct peaks at ∼400 and 800 nm close to the edges of the visible spectrum and are similar to those observed for CaCu_3_Ti_4_O_12_.^58^ Interestingly, similar absorbance peaks on the edges of the visible spectrum were observed for Y_2_CuTiO_6_, which features a trigonal bipyramidal coordination of mixed Cu/Ti sites.^10^ The 800 nm peak is expected for octahedral Cu^2+^ with a *d*–*d* transition at around 1–1.5 eV.^59^ The spectrum of the mixed Y_10_Ni_0.5_Cu_0.5_Ti_4_O_24_ sample showed a combination of these features. The observed edges presumably arise from transitions into the vacant Ti 3*d* states from occupied Ni *d* and Cu *d* states, in agreement with the calculations for CaCu_3_Ti_4_O_12_.^58^ The difference in edge position correlates with the observed values of band gaps, which are narrowest for Y_10_CuTi_4_O_24_ (higher lying occupied *d* states of Cu) to widest for Y_10_NiTi_4_O_24_ (lower-lying occupied d states of Ni). The band gap values were extracted using the Tauc plot method (Figure 9b–d), as described by Makuła et al.^26^ Y_10_CuTi_4_O_24_ and Y_10_Ni_0.5_Cu_0.5_Ti_4_O_24_ are characterized with band gaps of 2.713(5) eV and 2.715(3) eV, respectively. These values are lower than the reported band gap of 3.6 eV^60^ for Y_2_CuTiO_6_. At the same time, Y_10_NiTi_4_O_24_ is characterized by a band gap of 3.537(4) eV, which is higher than the values observed for Y_2_NiTiO_6_ (2.16–2.239 eV).^6^

**Figure 9:**
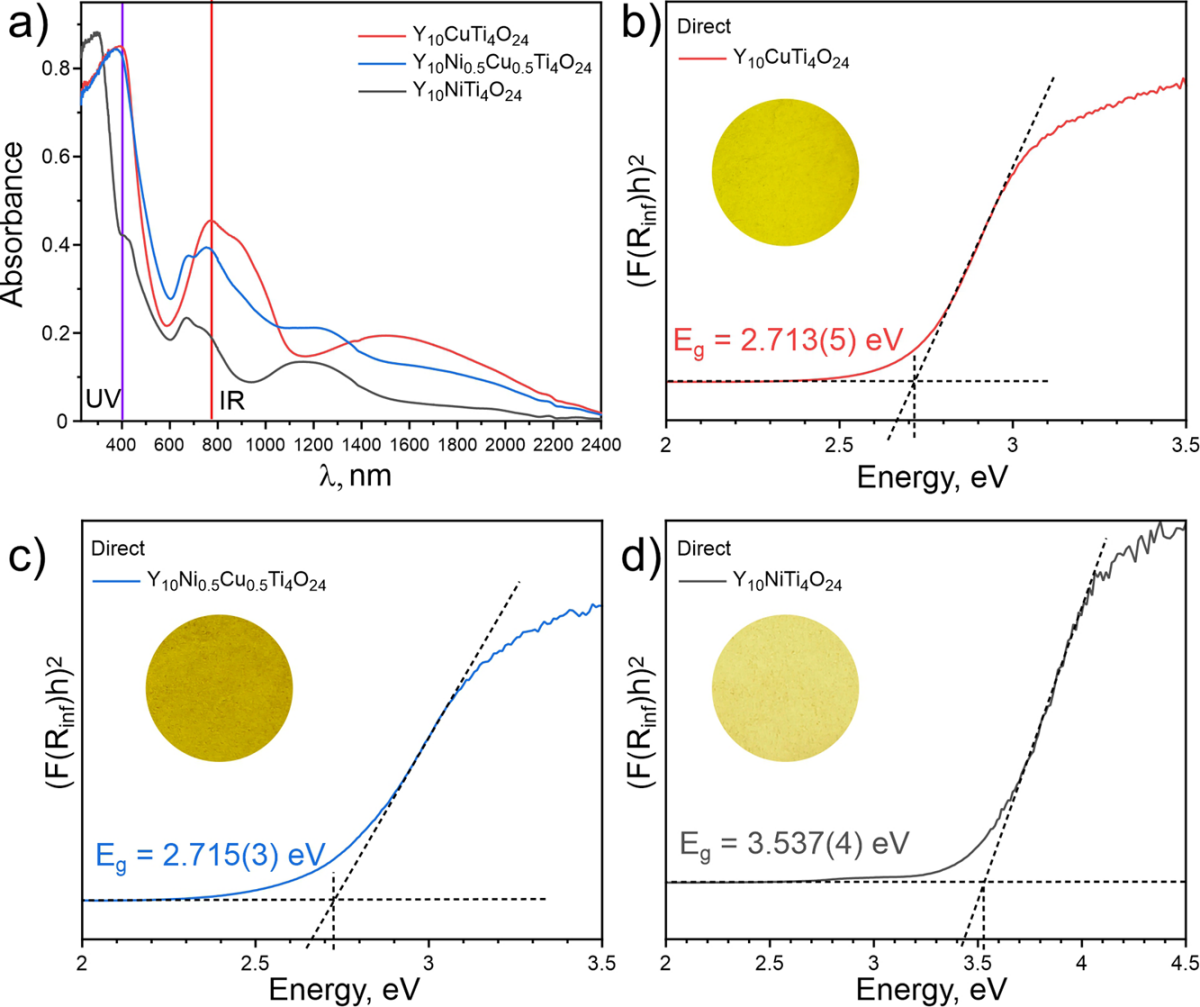
(a) Absorbance spectra of Y_10_Ni_*x*_Cu_1–*x*_Ti_4_O_24_ samples (*x* = 0, 0.5, 1, drawn in red, blue, and gray, respectively). Reflectance spectra of Y_10_CuTi_4_O_24_ (b), Y_10_Ni_0.5_Cu_0.5_Ti_4_O_24_ (c), and Y_10_NiTi_4_O_24_ (d) were used to determine direct optical band gaps for these phases using the Tauc plot method, as described by Makuła et al.^26^ Insets in (b–d) show the colors of respective samples.

## 4 Conclusions

The discovery of the Y_10_Ni_*x*_Cu_1–*x*_Ti_4_O_24_ (*x* = 0, 0.5, 1) phases through investigation of the structural boundary in the Y_2_NiTiO_6_–Y_2_CuTiO_6_ system demonstrates that new structures can be found at the compositional interfaces between known structure types. Interestingly, the new phase moved toward the interface between the starting oxides Y_2_O_3_ and TiO_2_ and even shares structural fragments with the latter. The structure of Y_10_Ni_*x*_Cu_1–*x*_Ti_4_O_24_ (*x* = 0, 0.5, 1) follows the general layered motif of the known Y_5_Mo_2_O_12_ structure type, with one striking difference: it contains an extra site occupied by Ni/Cu that is vacant in the structure of the molybdate. The structures of three Y_10_Ni_*x*_Cu_1–*x*_Ti_4_O_24_ (*x* = 0, 0.5, 1) members are similar; the only difference arises in the coordination of the Ni/Cu site that exhibits Jahn–Teller distortion for the samples containing Cu. Due to the low content of Cu/Ni, the three phases show Curie–Weiss paramagnetism with μ_eff_ = 2.148(3) μ_B_ for Y_10_CuTi_4_O_24_, 3.063(1) μ_B_ for Y_10_Ni_0.5_Cu_0.5_Ti_4_O_24_, and 3.251(1) μ_B_ for Y_10_NiTi_4_O_24_. The smaller content of Cu/Ni (3.58 at%) also explains the light-yellow color of this phase, which contrasts with green Y_2_NiTiO_6_ and Y_2_CuTiO_6_. Despite this minor first transition series content, just one atom out of 39 in the formula unit, the occupation of the extra octahedral site by these ions to form a rutile layer absent in the parent Y_5_Mo_2_O_12_ structure allows the new quaternary phases to be isolated despite competition with well-known stable ternaries in the phase field, such as pyrochlore Y_2_Ti_2_O_7_.

## Supplementary Material



## Notes

Additional crystallographic information on the Y_10_Ni_*x*_Cu_1–*x*_Ti_4_O_24_ (*x* = 0, 0.5, 1) phases; details on the stacking fault model used to fit the PXRD data of the Y_10_CuTi_4_O_24_ sample; Topas input file used for 1-dimentional grid search for the stacking fault model of Y_10_CuTi_4_O_24_; Topas input file used to fit the stacking fault model of Y_10_CuTi_4_O_24_; and additional measurement data of the Y_10_Ni_*x*_Cu_1–*x*_Ti_4_O_24_ (*x* = 0, 0.5, 1) phases (PDF)
