# Supplementary material for: Discovery at the Interface between Known Structure Types: The Layered Y10Ni x Cu1–x Ti4O24 Family
Source: Inorg Chem. 2026 Jul 12;65(29):17065–76. doi: 10.1021/acs.inorgchem.6c02278 (PMC13418174; doi:10.1021/acs.inorgchem.6c02278)
Supplement: Supplementary file 1 [file ic6c02278_si_001.pdf]

Discovery at the interface between known structure types: the layered $Y_{10}Ni_xCu_{1-x}Ti_4O_{24}$ family

Authors:

Nataliya L. Gulay[1], Hai Lin[1], Batoul Almoussawi[1], Cara J. Hawkins[1], Manel Sonni[1], Marco Zanella[1], Troy D. Manning,[1] Luke M. Daniels[1], Matthew S. Dyer[1], John B. Claridge[1], Matthew J. Rosseinsky*[1,2]

1. Department of Chemistry, University of Liverpool, Materials Innovation Factory, 51 Oxford Street, Liverpool, L7 3NY, UK

2. Leverhulme Research Centre for Functional Materials Design, Materials Innovation Factory, 51 Oxford Street, University of Liverpool, Liverpool L7 3NY, UK.

Supplementary information

SI1	Additional crystallographic information on the $Y_{10}Ni_xCu_{1-x}Ti_4O_{24}$ (*x* = 0, 0.5, 1) phases	2
SI2	Details on stacking faults model used to fit PXRD data of the $Y_{10}CuTi_4O_{24}$ sample	9
SI3	Topas input file used for 1-dimentional grid search for stacking-faulted model of $Y_{10}CuTi_4O_{24}$	11
SI4	Topas input file used to fit stacking-faulted model of $Y_{10}CuTi_4O_{24}$	15
SI5	Additional measurement data of the $Y_{10}Ni_xCu_{1-x}Ti_4O_{24}$ (*x* = 0, 0.5, 1) phases	19
	References	19

SI1. Additional crystallographic information on the $Y_{10}Ni_xCu_{1-x}Ti_4O_{24}$ (x = 0, 0.5, 1) phases

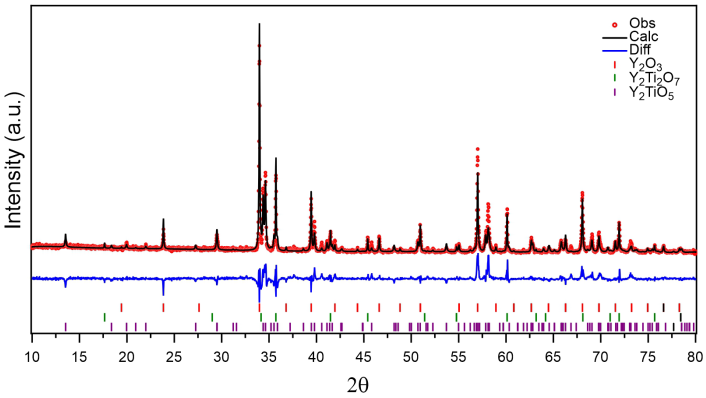

Figure SI1. In-house PXRD data (Cu Kα radiation) of the sample with the composition of $Y_{68.1}Ti_{23.3}O_{148.75}$ (Ni-and Cu-free analogue of the sample where first $Y_{10}Ni_xCu_{1-x}Ti_4O_{24}$ phase was discovered) which formed a mixture of Y_2O_3, $Y_2Ti_2O_7$, and Y_2TiO_5 while at the analogous conditions as those used to prepare $Y_{10}Ni_{0.5}Cu_{0.5}Ti_4O_{24}$. Observed, calculated, and the difference intensity are drawn in red circles, black line, and blue line, respectively.

Table SI1. Site coordinates and displacement parameters (Å^2) for $Y_{10}CuTi_4O_{24}$ (space group *C*2/*m*, *Z* = 1) from I19 (λ = 0.6889 Å) single diffraction data collected at 100K. The equivalent isotropic displacement parameter U_{eq} is defined as U_{eq} = 1/6ΣU_{ij}.

Site	Wyck.	*x/a*	*y/b*	*z/c*	U_{11}	U_{22}	U_{33}	U_{12}	U_{13}	U_{23}	U_{eq}
0.5O1a	8*j*	0.1353(6)	0.234(1)	0.062(1)	–	–	–	–	–	–	0.0052(7)
0.5O1b	8*j*	0.1569(6)	0.250(1)	0.075(1)	–	–	–	–	–	–	0.0052(7)
O2	8*j*	0.3356(2)	0.2658(5)	0.4132(4)	0.008(1)	0.003(1)	0.007(1)	–0.0003(9)	0.0046(9)	–0.0003(8)	0.0052(7)
O3	4*i*	0.0010(3)	0	0.3122(5)	0.007(2)	0.003(2)	0.007(1)	0	0.003(1)	0	0.005(1)
Y1	4*i*	0.19661(4)	0	0.34502(7)	0.0051(2)	0.0008(2)	0.0063(2)	0	0.0043(2)	0	0.0035(1)
O4	4*i*	0.5024(3)	0	0.3202(5)	0.011(2)	0.003(2)	0.005(1)	0	0.005(1)	0	0.006(1)
Y2	4*i*	0.80214(4)	0	0.14705(6)	0.0058(2)	0.0010(2)	0.0041(2)	0	0.0022(2)	0	0.0035(1)
Ti1	4*h*	0	0.2600(2)	1/2	0.0047(4)	0.0009(4)	0.0042(3)	0	0.0028(3)	0	0.0030(2)
0.5Y3	4*g*	0	0.4718(4)	0	0.0043(3)	0.003(1)	0.0034(3)	0	0.0029(2)	0	0.0032(5)
0.509(4)Cu1	2*a*	0	0	0	0.0049(8)	0.0043(8)	0.0047(8)	0	0.0026(6)	0	0.0044(5)

Table SI2. Complete list of interatomic distances (Å) for the atoms in the structure of $Y_{10}CuTi_4O_{24}$ (space group *C*2/*m*, *Z* = 1) from I19 (λ = 0.6889 Å) single diffraction data collected at 100K. Standard deviations are equal or smaller than 0.003Å.

Atoms			*d*, Å	Atoms			*d*, Å	Atoms			*d*, Å	Atoms			*d*, Å
O1a	1x	Cu1	2.094	O2	1x	Ti1	1.929	Y1	2x	O2	2.252	Y3	2x	O1a	2.112
	1x	Y3	2.112		1x	Y1	2.253		2x	O2	2.335		2x	O1b	2.249
	1x	Y2	2.322		1x	Y2	2.289		1x	O3	2.337		2x	O4	2.294
	1x	Y3	2.343		1x	Y1	2.335		2x	O1b	2.364		2x	O1a	2.344
	1x	Y1	2.379	O3	2x	Ti1	2.037		2x	O1a	2.379		2x	O1b	2.454
	1x	Y2	2.500		1x	Cu1	2.236	Y2	2x	O1b	2.244	Ti1	2x	O4	1.915
O1b	1x	Y2	2.244		1x	Y1	2.336		2x	O2	2.289		2x	O2	1.930
	1x	Y3	2.2492		1x	Y2	2.374		2x	O1a	2.321		2x	O3	2.037
	1x	Y2	2.326	O4	2x	Ti1	1.915		2x	O1b	2.325		1x	Ti1	2.814
	1x	Cu1	2.350		2x	Y3	2.294		1x	O3	2.373		1x	Ti1	3.049
	1x	Y1	2.364						2x	O1a	2.500	Cu1	4x	O1a	2.094
	1x	Y3	2.455										2x	O3	2.236
													4x	O1b	2.350

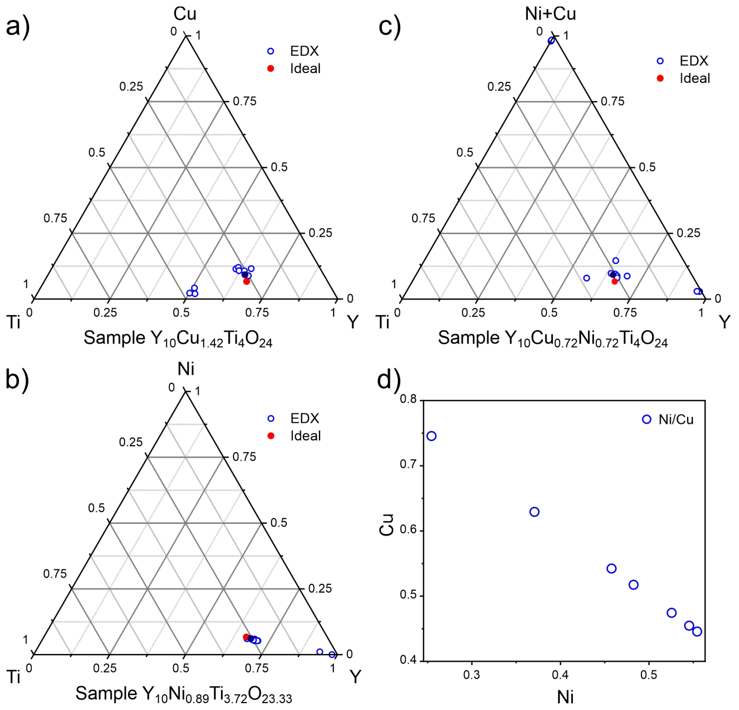

Figure SI2. Results of the EDX analysis of polycrystalline $Y_{10}Ni_xCu_{1-x}Ti_4O_{24}$ (x = 0, 0.5, 1) samples (a-c). Starting nominal compositions are given below while ideal 10:1:4 composition corresponding to refined formula are marked with a red circle. The outlier points correlate well with by-products consistent with results of PXRD analysis (Table SI4). (d) shows distribution of Ni/Cu content in the $Y_{10}Cu_{0.72}Ni_{0.72}Ti_4O_{24}$ sample.

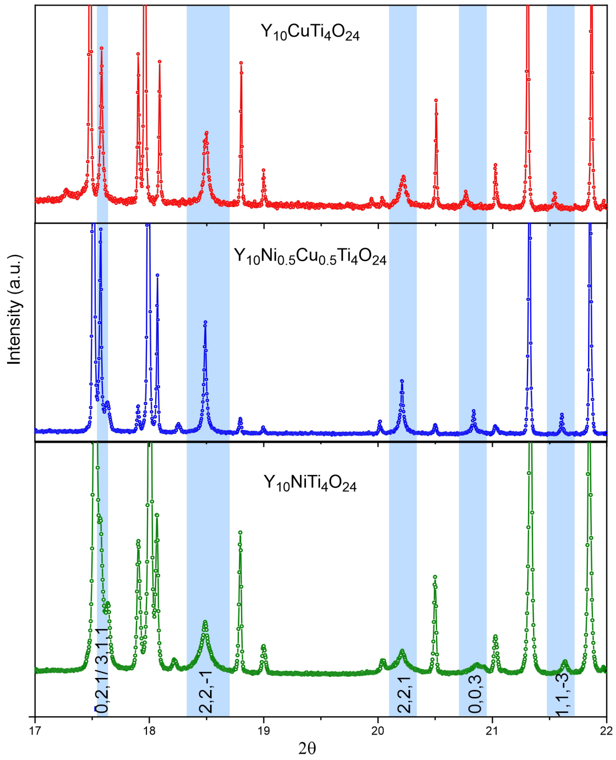

Figure SI3. Comparison of the synchrotron PXRD data (λ = 0.824672 Å) collected at room temperature (298 K) $Y_{10}Ni_xCu_{1-x}Ti_4O_{24}$ (x = 0, 0.5, 1) phases (space group $C2/m$, Z = 1). The broader peaks with $l = 2n+1$ are highlighted. $Y_{10}CuTi_4O_{24}$, $Y_{10}Ni_{0.5}Cu_{0.5}Ti_4O_{24}$, and $Y_{10}NiTi_4O_{24}$ samples are shown in top, middle, and bottom panels, respectively.

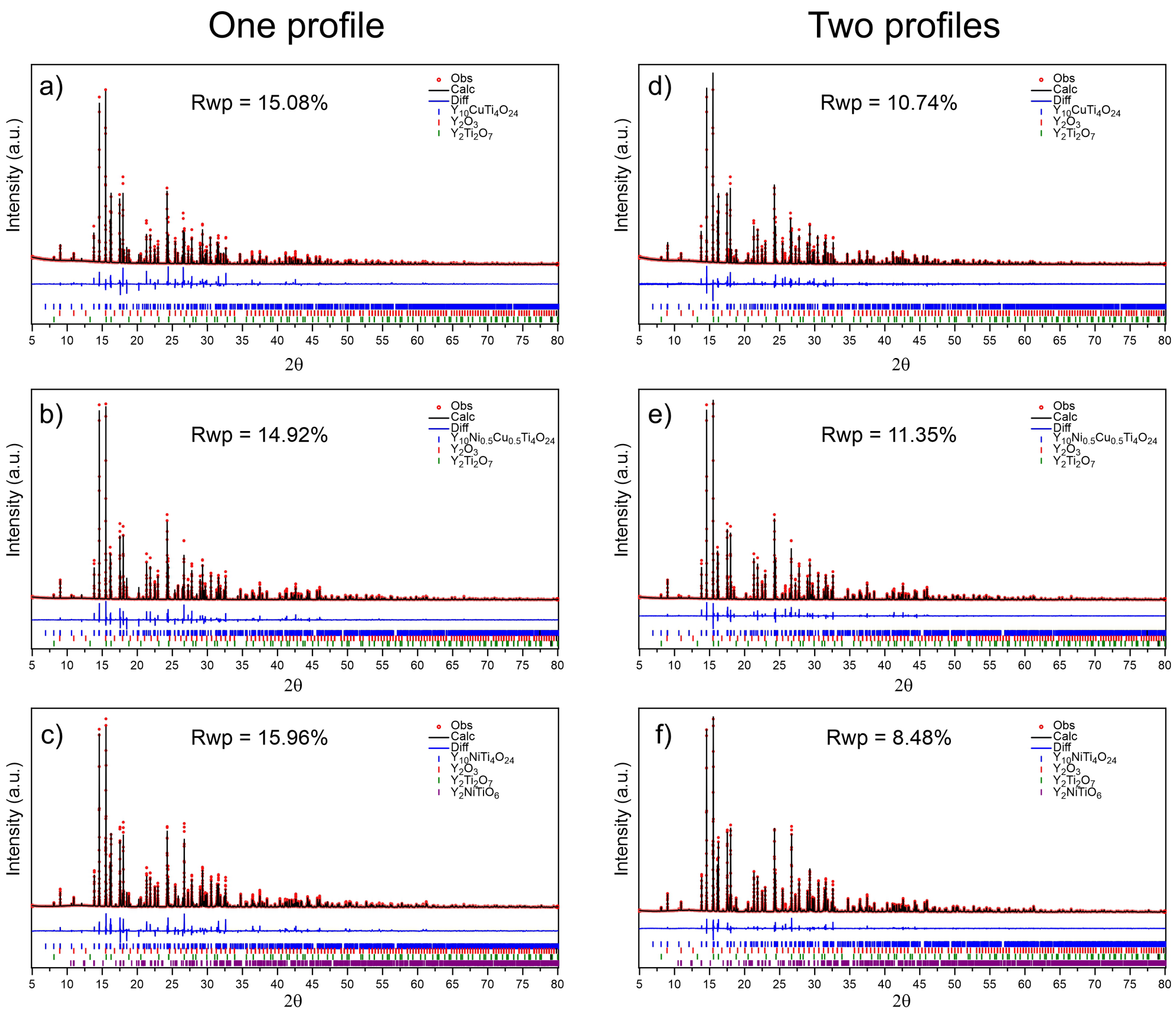

Figure SI4. Comparison of Rietveld refinement against the synchrotron PXRD data (λ = 0.824672 Å) collected at room temperature (298 K) using one (a-c) versus two (d-f) profile functions for $Y_{10}Ni_xCu_{1-x}Ti_4O_{24}$ (x = 0, 0.5, 1) phases (space group *C*2/*m*, *Z* = 1). $Y_{10}CuTi_4O_{24}$, $Y_{10}Ni_{0.5}Cu_{0.5}Ti_4O_{24}$, and $Y_{10}NiTi_4O_{24}$ samples are shown in top, middle, and bottom panels, respectively. Quantities of phases based on two-profile model refinement is given in Table SI4. Observed, calculated, and the difference intensity are drawn in red circles, black line, and blue line, respectively.

Table SI3. Crystallographic data and refinement details for $Y_{10}Ni_xCu_{1-x}Ti_4O_{24}$ (x = 0, 0.5, 1) phases (space group $C2/m$, Z = 1) from Rietveld refinements using two profile function model of the synchrotron PXRD data (λ = 0.824672 Å) collected at room temperature (298 K). L_{even} and L_{odd} correspond to volume weighted crystallite size peak broadening[1] for peaks with l=2n and l=2n+1, respectively.

Phase	$Y_{10}NiTi_4O_{24}$	$Y_{10}Ni_{0.5}Cu_{0.5}Ti_4O_{24}$	$Y_{10}CuTi_4O_{24}$
Formula weight, g mol^{-1}	1523.21	1525.63	1528.06
Lattice parameters, Å	a = 12.24649(6)<br>b = 5.87759(2)<br>c = 7.14454(2)<br>β = 106.9683(8)°	a = 12.24609(3)<br>b = 5.87615(1)<br>c = 7.15521(2)<br>β = 107.0169(2)°	a = 12.24513(3)<br>b = 5.86987(1)<br>c = 7.15164(2)<br>β = 107.0645(3)°
Cell volume, Å^3	491.876(4)	492.345(2)	493.471(2)
Density, g cm^{-3}	5.14224(4)	5.14553(2)	5.14194(2)
Sample contents, wt. %			
$Y_{10}Ni_xCu_{1-x}Ti_4O_{24}$	81.37(7)	96.04(5)	81.66(9)
Y_2O_3	7.73(5)	2.38(5)	8.47(7)
$Y_2Ti_2O_7$	7.37(3)	1.59(3)	9.87(6)
Y_2NiTiO_6	3.54(5)		
2θ, ° range	5–80	5–80	5–80
2θ, ° step	0.004	0.004	0.004
No. of refined parameters	99	80	80
R_p, %	6.16	7.95	7.75
R_{wp}, %	8.49	11.34	10.74
R_{exp}, %	1.97	3.16	4.37
R_{Bragg}, %	2.27	3.99	5.14
GOF (R_{wp}/R_{exp})	4.32	3.59	2.46
L_{even}, nm	522(56)	746(104)	1559(133)
L_{odd}, nm	30(2)	128(3)	73(3)
L_{odd} : L_{even}	1 : 17.4	1 : 5.8	1 : 21.2

Table SI4. Site coordinates and isotropic thermal parameters for $Y_{10}Ni_xCu_{1-x}Ti_4O_{24}$ (x = 0, 0.5, 1) (space group *C*2/*m*, *Z* = 1) from Rietveld refinements of the synchrotron PXRD data (λ = 0.824672 Å) collected at room temperature.

Site	Wyck.	*x/a*	*y/b*	*z/c*	B_{iso}, Å^2
		$Y_{10}NiTi_4O_{24}$			
0.5O1a	8*j*	0.142(1)	0.244(2)	0.062(4)	0.9(1)
0.5O1b	8*j*	0.161(1)	0.250(3)	0.086(4)	0.9(1)
O2	8*j*	0.3409(3)	0.267(1)	0.415(2)	0.11(8)
O3	4*i*	0.003(1)	0	0.3106(8)	0.45(8)
Y1	4*i*	0.19666(9)	0	0.3456(4)	0.41(2)
O4	4*i*	0.502(1)	0	0.3199(8)	0.14(7)
Y2	4*i*	0.80061(9)	0	0.1444(4)	0.33(2)
Ti1	4*h*	0	0.2605(2)	1/2	0.46(2)
0.5Y3	4*g*	0	0.4731(3)	0	0.47(3)
0.5Ni1	2*a*	0	0	0	0.48(4)
		$Y_{10}Ni_{0.5}Cu_{0.5}Ti_4O_{24}$			
0.5O1a	8*j*	0.1365(7)	0.229(2)	0.062(2)	0.25(9)
0.5O1b	8*j*	0.1598(8)	0.253(2)	0.080(2)	0.25(9)
O2	8*j*	0.3386(3)	0.2673(8)	0.4162(8)	0.32(7)
O3	4*i*	0.0025(7)	0	0.3106(7)	0.8(1)
Y1	4*i*	0.19689(8)	0	0.3442(2)	0.33(2)
O4	4*i*	0.5021(7)	0	0.3203(7)	0.37(8)
Y2	4*i*	0.80105(7)	0	0.1457(2)	0.30(2)
Ti1	4*h*	0	0.2601(2)	1/2	0.50(2)
0.5Y3	4*g*	0	0.4729(4)	0	0.41(3)
0.25Ni1+ 0.25Cu1	2*a*	0	0	0	0.61(5)
		$Y_{10}CuTi_4O_{24}$			
0.5O1a	8*j*	0.138(1)	0.226(3)	0.068(4)	0.9(2)
0.5O1b	8*j*	0.165(1)	0.257(3)	0.079(4)	0.9(2)
O2	8*j*	0.3404(5)	0.271(1)	0.413(2)	0.7(1)
O3	4*i*	0.001(1)	0	0.314(1)	0.9(1)
Y1	4*i*	0.1964(1)	0	0.3459(4)	0.73(3)
O4	4*i*	0.506(1)	0	0.3188(9)	0.2*
Y2	4*i*	0.8026(1)	0	0.1454(4)	0.77(3)
Ti1	4*h*	0	0.2597(3)	1/2	0.81(3)
0.5Y3	4*g*	0	0.4713(5)	0	0.94(4)
0.5Cu1	2*a*	0	0	0	1.11(7)

**fixed during refinement*

Table SI5. Selected interatomic distances (Å) for the atoms in the structure of $Y_{10}Ni_xCu_{1-x}Ti_4O_{24}$ (x = 0, 0.5, 1) (space group $C2/m$, Z = 1) from Rietveld refinements of the synchrotron PXRD data (λ = 0.824672 Å) collected at room temperature. Standard deviations are equal or smaller than 0.007Å.

$Y_{10}NiTi_4O_{24}$				$Y_{10}Ni_{0.5}Cu_{0.5}Ti_4O_{24}$				$Y_{10}CuTi_4O_{24}$			
Atoms			*d*, Å	Atoms			*d*, Å	Atoms			*d*, Å
Y1	2x	O1b	2.306	Y1	2x	O2	2.285	Y1	2x	O2	2.318
	2x	O2	2.307		1x	O3	2.321		2x	O1a	2.328
	1x	O3	2.315		2x	O2	2.331		1x	O3	2.337
	2x	O2	2.336		2x	O1b	2.343		2x	O2	2.340
	2x	O1a	2.413		2x	O1a	2.358		2x	O1b	2.380
Y2	2x	O1b	2.197	Y2	2x	O1b	2.201	Y2	2x	O1b	2.155
	2x	O2	2.304		2x	O1a	2.295		2x	O2	2.277
	2x	O1a	2.312		2x	O2	2.304		2x	O1a	2.301
	2x	O1b	2.352		2x	O1b	2.345		2x	O1b	2.326
	2x	O1a	2.395		1x	O3	2.403		1x	O3	2.374
	1x	O3	2.412		2x	O1a	2.501		2x	O1a	2.512
Y3	2x	O1a	2.214	Y3	2x	O1a	2.148	Y3	2x	O1a	2.163
	2x	O4	2.283		2x	O1b	2.274		2x	O4	2.275
	2x	O1b	2.300		2x	O4	2.290		2x	O1b	2.302
	2x	O1a	2.348		2x	O1a	2.372		2x	O1a	2.400
	2x	O1b	2.494		2x	O1b	2.469		2x	O1b	2.502
Ti1	2x	O2	1.871	Ti1	2x	O2	1.897	Ti1	2x	O2	1.877
	2x	O4	1.912		2x	O4	1.913		2x	O4	1.935
	2x	O3	2.050		2x	O3	2.049		2x	O3	2.029
	1x	Ti1	2.815		1x	Ti1	2.819		1x	Ti1	2.821
	1x	Ti1	3.062		1x	Ti1	3.057		1x	Ti1	3.049
Ni1	4x	O1a	2.192	Ni1/Cu1	4x	O1a	2.089	Cu1	4x	O1	2.089
	2x	O3	2.210		2x	O3	2.214		2x	O3	2.253
	4x	O1b	2.394		4x	O1b	2.389		4x	O1b	2.448

SI2. Details on stacking faults model used to fit PXRD data of the $Y_{10}CuTi_4O_{24}$ sample

While the two-phase model provided a much better fit to the experimental data, some discrepancies in the fits of the *l*-odd peaks remained. Upon careful examination we realized that there is a range of peak shapes for reflections that didn't follow any clear *hkl* rules which is the most evident from the $Y_{10}CuTi_4O_{24}$ composition (see sharper (021), (311), (22–3), (51–3) and broader (22–1), (221), (42–1) peaks in Figure 5, b). Similar features are seen in the data from $Y_{10}NiTi_4O_{24}$ but they are less visible due to overlap from the secondary phase peaks. Given that related $RE_5Ru_2O_{12}$ (*RE* = Pr, Nd, Sm–Tb) compounds exhibited stacking faults seen from the high-resolution transition electron microscopy,[2] we developed a model to account for possible stacking faults arising along the *c*-axis was developed and refined for $Y_{10}CuTi_4O_{24}$. The stacking faults probability search and fitting were done with Topas V7 using a methodology applied for the $Li_3HoBr_{6-x}I_x$[3] and $Ag_3LiRu_2O_6$[4] phases.

The stacking faults probability search and fitting were done with Topas V7 using a methodology applied for the $Li_3HoBr_{6-x}I_x$.[3] For this, the model was transformed from *C*2/*m* to the *P*1 space group and oriented so the monoclinic *a*-axis became *c*-axis in the triclinic model. Constraints were applied to the equivalent atomic sites to preserve the pseudo-monoclinic setting, similar to how it was done for fitting of $Ag_3LiRu_2O_6$.[4] The stacking was described as a probability of the unit cell to translate into the equivalent cell, shifted by ½ along the original monoclinic *c*-axis, within 100 stacks along the original monoclinic a-axis. Completely faultless structure would have this stacking probability of 0 while a value of 1 would indicate shifts after each translation. Initial test showed a small *p* value under 0.1; therefore, the number of stacks was increased to 200 to increase refinement accuracy. To find the optimal stacking probability (*p*), the 1-dimentional grid search was run[4] and averaged for 10 sequences of 200 stacks, and the obtained values of R_{wp} were plotted against the stacking probability (Figure SI5). The minimal value of R_{wp} was obtained for *p* of 0.041 which was used for the refinement of the model, and the final run was done using 50 sequences of 200 stacks.

The refinement of the structural model with *p*=0.041 improves the statistics and shows improved fit to the experimental data correctly describing uniformity in intensities and peak shape (see Figure 5). At the same time, statistics improvement was less dramatic which is expected given that these fine features arise from very small peaks on the synchrotron PXRD (the right panel in Figure 5 shows PXRD regions enlarged ~ 5 times).

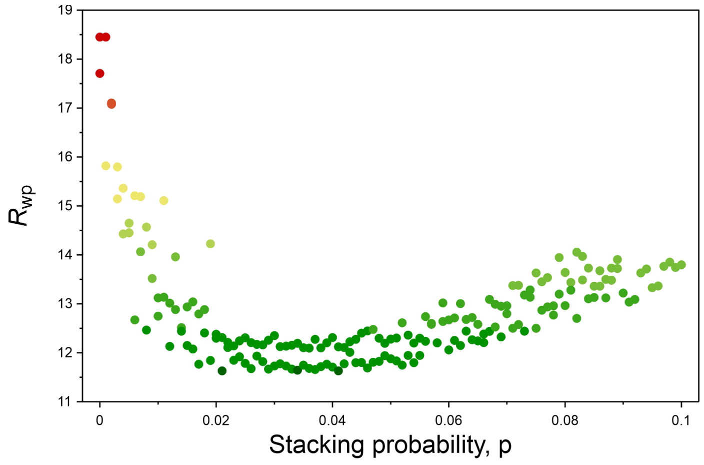

Figure SI5. 1-dimentional grid search for the stacking probability for the $Y_{10}CuTi_4O_{24}$ sample

Table SI6. Pseudo-monoclinic restraints on the site coordinates within the *P*1 model for $Y_{10}CuTi_4O_{24}$ (real space group *C*2/*m*, *Z* = 1) used to fit stacking-faulted model with Topas V7.0 (input file is provided as separate supplementary information). O1a/O1b splitting was omitted to minimize the number of refined parameters. N_v stands for the number of stacks per sequence, which was 200 for the final refinement. Constrains labelled as the corresponding coordinates in the structure of the monoclinic $Y_{10}CuTi_4O_{24}$.

*C*2/*m* model		*P*1 model			
Site	Wyck.	Site	*x/a*	*y/b*	*z/c*
O1	8*j*	O1	O1z	O1y	O1x/N_v
		O1002	–O1z	O1y	–O1x/N_v
		O1003	–O1z	–O1y	–O1x/N_v
		O1004	O1z	–O1y	O1x/N_v
		O1005	O1z	O1y + ½	(O1x+ ½)/N_v
		O1006	–O1z	O1y + ½	(–O1x+ ½)/N_v
		O1007	–O1z	–O1y + ½	(O1x+ ½)/N_v
		O1008	O1z	–O1y + ½	(O1x+ ½)/N_v
O2	8*j*	O2	O2z	O2y	O2x/N_v
		O2002	–O2z	O2y	–O2x/N_v
		O2003	–O2z	–O2y	–O2x/N_v
		O2004	O2z	–O2y	O2x/N_v
		O2005	O2z	O2y + ½	(O2x+ ½)/N_v
		O2006	–O2z	O2y + ½	(–O2x+ ½)/N_v
		O2007	–O2z	–O2y + ½	(O2x+ ½)/N_v
		O2008	O2z	–O2y + ½	(O2x+ ½)/N_v
O3	4*i*	O3	O3z	0	O3x/N_v
		O3002	–O3z	0	–O3x/N_v
		O3003	O3z	½	(O3x+ ½)/N_v
		O3004	–O3z	½	(–O3x+ ½)/N_v
Y1	4*i*	Y1	Y1z	0	Y1x/N_v
		Y1002	–Y1z	0	–Y1x/N_v
		Y1003	Y1z	½	(Y1x+ ½)/N_v
		Y1004	–Y1z	½	(–Y1x+ ½)/N_v
O4	4*i*	O4	O4z	0	O4x/N_v
		O4002	–O4z	0	–O4x/N_v
		O4003	O4z	½	(O4x+ ½)/N_v
		O4004	–O4z	½	(–O4x+ ½)/N_v
Y2	4*i*	Y2	Y2z	0	Y2x/N_v
		Y2002	–Y2z	0	–Y2x/N_v
		Y2003	Y2z	½	(Y2x+ ½)/N_v
		Y2004	–Y2z	½	(–Y2x+ ½)/N_v
Ti1	4*h*	Ti1	½	Ti1y	0/N_v
		Ti1002	½	–Ti1y	0/N_v
		Ti1003	½	–Ti1y + ½	½/N_v
		Ti1004	½	Ti1y + ½	½/N_v
0.5Y3	4*g*	Y3	0	Y3y	0/N_v
		Y3002	0	–Y3y	0/N_v
		Y3003	0	–Y3y + ½	½/N_v
		Y3004	0	Y3y + ½	½/N_v
0.5Cu1	2*a*	Cu1	0	0	0/N_v
		Cu1002	0	½	½/N_v

SI3. Topas input file used for 1-dimentional grid search for stacking-faulted model of $Y_{10}CuTi_4O_{24}$

```
'=== FILE HEADER ===
r_wp  12.7409293 r_exp  5.16511519 r_p  9.18366928 r_wp_dash  13.8686051 r_p_dash  10.7104656
r_exp_dash  5.62226984 weighted_Durbin_Watson  0.876979485 gof  2.46672705
iters 100000 'Maximum number of iterations of refinement
chi2_convergence_criteria 0.001 'Stop criteria for refinement

'  Number of steps in the grid
num_runs 101

'=== POWDER DIFFRACTION FILE ===
xdd 1323544_0004_summed_0004.xye

'=== FILE PREPARATION ===
x_calculation_step = Yobs_dx_at(Xo); 'Sets the calculation step size for Rietveld refinement.

'   Instrumental parameters and features below are fixed based on best fit from non-stacked model

'=== BACKGROUND FUNCTION ===
bkg  66.8119114` -62.2322076`  90.4393238` -131.421821`  145.619484` -129.145715`
    88.3556163` -42.6304976`  12.4693381`  5.26807435` -6.58621341`  4.67183759`

   PV(,,,, 16351.1494` , 11.295, 12.4388062` , 2.03035064e-15` _LIMIT_MIN_0)

'=== RADIATION SOURCE - Information on the profile of a synchrotron X-ray source ===
Simple_Axial_Model(!axial, 1.94549162` ) 'Describes peak asymmetry due to axial divergence
LP_Factor( ,90.0) 'Lorentz-Polarisation factor. Change the monochromator angle as required
lam ymin_on_ymax 0.0001 la 1.0 lo 0.824672 lh 0.1 'Radiation profile

            Specimen_Displacement(,-0.00693312781` )

start_X  5
finish_X  80

 '=== Structure with the stacking faults in P1 setting, oriented so stacking occurs along c-axis  ===

                        str

                        phase_name "Y10CuTi4O24"
                        a  = lpc;
                        b  = lpb;
                        c  = Get(generated_c);

'=== Original cell in monoclinic setting, for reference
                        prm !lpa  12.2453344`
                        prm !lpb  5.86991633`
                        prm !lpc  7.18167511`

                        al          90
                        be    72.93595`    ' inverted from monoclinic be @  107.06298`
                        ga          90
```

```
			space_group "P1"

prm !px = (##Run_Number##/1000);		'defines step for p, corresponds to 0.1%, and with number of runs 101 it will amount to calculating p from 0 to 10%

out "1-dimensional-grid_shorter_step.txt" append
Out(Get(r_wp),"\t%11.5f")
Out(px, "\t%11.5f\n")

			prm s  1.25132899`  min 1e-15
			scale = s 1e-6/ (Nv Nstr);

		generate_stack_sequences {
			pr_str { B layers_tol} 10
			number_of_sequences Nstr 10
			number_of_stacks_per_sequence Nv 200	' increased from 100 due to small value of p
				Transition(A, lpa)
to A = 1-px;		a_add =  0;  b_add = 0;		'translation in the same cell
to B = px;		 a_add =  1/2;  b_add =  0;	'translation in the cell shifted by 1/2 along a in P1 setting
				Transition(B, A, lpa)
		to A = 1-px;		a_add =  0;  b_add = 0;
		to B = px;		a_add =  1/2;  b_add = 0;

'=== Atomic sites as refined in the monoclinic model===

			prm  !O1x  0.851510164`
			prm  !O1y  0.234474382`
			prm  !O1z  0.0704968147`
			prm  !O1beq  1.29307014`		min 0 max 5

			prm  !O2x  0.661917113`
			prm  !O2y  0.263941721`
			prm  !O2z  0.411780432`
			prm  !O2beq  0.659899671`_LIMIT_MIN_0		min 0 max 5

			prm  !O3x -0.00636808455`
			prm  !O3z  0.317112386`
			prm  !O3beq  0.724547666`_LIMIT_MIN_0		min 0 max 5

			prm  !Y1x  0.80332298`
			prm  !Y1z  0.347317551`
			prm  !Y1beq  0.765928008`		min 0 max 5

			prm  !O4x  0.494333154`
			prm  !O4z  0.318478248`
			prm  !O4beq  0.00463240116`_LIMIT_MIN_0	min 0 max 5

			prm  !Y2x  0.197149804`
			prm  !Y2z  0.145772823`
			prm  !Y2beq  0.68891541`		min 0 max 5

			prm  !Y3beq  1.93379154`		min 0 max 5
```

```
		prm  !Ti1y  0.261361389`
		prm  !Ti1beq  0.930900241`		min 0 max 5

		prm  !Cu1occ  0.5		min 0 max 1
		prm  !Cu1beq  1.83453206`		min 0 max 5
		volume  98615.511

'===Restricted atomic sites to preserve initial monoclinic symmetry===

site O1    x  =  (O1z);   y = O1y;       z = (O1x)/Nv;           occ O    1    beq = O1beq;          layer A
site O1002  x  =  (-O1z);  y = O1y;       z = (-O1x)/Nv;           occ O    1    beq = O1beq;       layer A
site O1003  x  =  (-O1z);  y = -O1y;      z = (-O1x)/Nv;           occ O    1    beq = O1beq;       layer A
site O1004  x  =  (O1z);   y = -O1y;      z = (O1x)/Nv;           occ O    1    beq = O1beq;       layer A
site O1005  x  =  (O1z);   y = O1y + 1/2;  z = (O1x + 1/2)/Nv;       occ O    1    beq = O1beq;            layer A
site O1006  x  =  (-O1z);  y = O1y + 1/2;  z = (-O1x + 1/2)/Nv;       occ O    1    beq = O1beq;            layer A
site O1007  x  =  (-O1z);  y = -O1y + 1/2; z = (-O1x + 1/2)/Nv;       occ O    1    beq = O1beq;            layer A
site O1008  x  =  (O1z);   y = -O1y + 1/2; z = (O1x + 1/2)/Nv;       occ O    1    beq = O1beq;            layer A
site O2    x  =  (O2z);    y = O2y;       z = (O2x)/Nv;           occ O    1    beq = O2beq;          layer A
site O2002  x  =  (-O2z);   y = O2y;       z = (-O2x)/Nv;          occ O    1    beq = O2beq;       layer A
site O2003  x  =  (-O2z);   y = -O2y;      z = (-O2x)/Nv;          occ O    1    beq = O2beq;       layer A
site O2004  x  =  (O2z);    y = -O2y;      z = (O2x)/Nv;           occ O    1    beq = O2beq;       layer A
site O2005  x  =  (O2z);    y = O2y + 0.5;  z = (O2x + 0.5)/Nv;       occ O    1    beq = O2beq;            layer A
site O2006  x  =  (-O2z);   y = O2y + 0.5;  z = (-O2x + 0.5)/Nv;       occ O    1    beq = O2beq;            layer A
site O2007  x  =  (-O2z);   y = -O2y + 0.5; z = (-O2x + 0.5)/Nv;      occ O    1    beq = O2beq;            layer A
site O2008  x  =  (O2z);    y = -O2y + 0.5; z = (O2x + 0.5)/Nv;       occ O    1    beq = O2beq;            layer A
site O3    x   =  (O3z);  y 0.00000      z = (O3x)/Nv;            occ O    1     beq = O3beq;       layer A
site O3002  x   =  (-O3z); y 0.00000      z = (-O3x)/Nv;           occ O    1     beq = O3beq;   layer A
site O3003  x   =  (O3z);  y = 1/2;       z = (O3x + 0.5)/Nv;       occ O    1     beq = O3beq;   layer A
site O3004  x   =  (-O3z); y = 1/2;       z = (-O3x + 0.5)/Nv;        occ O    1     beq = O3beq;  layer A
site Y1    x =  (Y1z);     y 0.00000      z = (Y1x)/Nv;           occ Y    1    beq = Y1beq;            layer A
site Y1002  x =  (-Y1z);    y 0.00000      z = (-Y1x)/Nv;          occ Y    1    beq = Y1beq;          layer A
site Y1003  x =  (Y1z);     y 0.50000      z = (Y1x + 0.5)/Nv;     occ Y    1    beq = Y1beq;         layer A
site Y1004  x =  (-Y1z);    y 0.50000      z = (-Y1x + 0.5)/Nv;     occ Y    1    beq = Y1beq;         layer A
site O4    x  =  (O4z);  y 0.00000      z = (O4x)/Nv;            occ O    1         beq = O4beq;    layer A
site O4002  x  =  (-O4z);  y 0.00000      z = (-O4x)/Nv;           occ O    1         beq = O4beq;    layer A
site O4003  x  =  (O4z);  y 0.50000      z = (O4x + 0.5)/Nv;       occ O    1         beq = O4beq;    layer A
site O4004  x  =  (-O4z);  y 0.50000      z = (-O4x + 0.5)/Nv;       occ O    1         beq = O4beq;    layer A
site Y2    x =  (Y2z);     y 0.00000      z = (Y2x)/Nv;         occ Y    1         beq = Y2beq;    layer A
site Y2002  x =  (-Y2z);    y 0.00000      z = (-Y2x)/Nv;        occ Y    1         beq = Y2beq;    layer A
site Y2003  x =  (Y2z);     y 0.50000      z = (Y2x + 0.5)/Nv;    occ Y    1         beq = Y2beq;    layer A
site Y2004  x =  (-Y2z);    y 0.50000      z = (-Y2x + 0.5)/Nv;    occ Y    1         beq = Y2beq;    layer A
site Ti1    x = 0.50000;  y = Ti1y;      z =0.00000/Nv;       occ Ti   1         beq = Ti1beq;    layer A
site Ti1002 x = 0.50000;   y = -Ti1y;      z =0.00000/Nv;       occ Ti   1         beq = Ti1beq;    layer A
site Ti1003 x = 0.50000;   y = -Ti1y +0.5; z =0.50000/Nv;       occ Ti   1         beq = Ti1beq;    layer A
site Ti1004 x = 0.50000;   y = Ti1y + 0.5; z =0.50000/Nv;       occ Ti   1         beq = Ti1beq;    layer A
site Y3    x 0.00000       y 0.50000       z 0.00000       occ Y    1                         beq = Y3beq;    layer A
site Y3002  x 0.00000       y 0.00000       z =0.50000/Nv;       occ Y    1         beq = Y3beq;     layer A
site Cu1    x 0.00000       y 0.00000       z 0.00000        occ Cu   = Cu1occ;       beq = Cu1beq;       layer A
site Cu1002 x 0.00000       y 0.50000       z =0.50000/Nv;         occ Cu   = Cu1occ;    beq = Cu1beq;      layer A

normalize_FCs
		peak_buffer_based_on = Xo;
		peak_buffer_based_on_tol 0.1
```

```
            PVII_Peak_Type(, 0.0001`_LIMIT_MIN_0.0001,, 0.0001`_LIMIT_MIN_0.0001,,
0.0248451041`,, 0.521961546`,, 0.204887983`,, 0.000100000001`_LIMIT_MIN_0.0001)

'=== structural information for inpurities, fixed all except scale according to the best fit
      str

            phase_name "Y2Ti2O7"
            Cubic(  10.099492`)
            space_group "Fd-3mz"
            scale @  5.65538563e-07`

            volume  1030.146`
            site O1    x  0.42864    y 0.125      z 0.125      occ O   1
            site Ti1   x 0.5        y 0.5        z 0.5        occ Ti   1
            site Y1    x 0          y 0          z 0          occ Y   1
            site O2    x 0.125      y 0.125      z 0.125      occ O   1

            PVII_Peak_Type(, 0.0001`_LIMIT_MIN_0.0001,, 0.0001`_LIMIT_MIN_0.0001,,
0.0226630806`,, 0.492012245`,, 0.0001`_LIMIT_MIN_0.0001,, 0.0001`_LIMIT_MIN_0.0001)

      str

            phase_name "Y2O3"
            Cubic(  10.602223`)
            space_group "206"
            scale @  4.38424427e-07`

            volume  1191.765`
            site O1    x 0.08347     y 0.37087     z 0.14708     occ O   1
            site Y1    x 0.28508     y 0           z 0.25        occ Y   1
            site Y2    x 0           y 0           z 0           occ Y   1

            PV_Peak_Type(, 0.0001`_LIMIT_MIN_0.0001,, 0.0733206627`,,
0.0001`_LIMIT_MIN_0.0001,, 0.8775`_LIMIT_MIN_0.0001,, 0.305`_LIMIT_MIN_0.0001,,
0.305`_LIMIT_MIN_0.0001)
```

SI4. Topas input file used to fit stacking-faulted model of $Y_{10}CuTi_4O_{24}$

```
'=== FILE HEADER ===
r_wp  10.3218385 r_exp  4.3726675 r_p  7.59272803 r_wp_dash  10.5797468 r_p_dash  8.2905304
r_exp_dash  4.48192585 weighted_Durbin_Watson  1.00346062 gof  2.36053589
iters 100000 'Maximum number of iterations of refinement
chi2_convergence_criteria 0.001 'Stop criteria for refinement

'=== POWDER DIFFRACTION FILE ===
xdd 1323544_0004_summed_0004.xye

'=== FILE PREPARATION ===
x_calculation_step = Yobs_dx_at(Xo); 'Sets the calculation step size for Rietveld refinement.

'   Instrumental parameters and features below are fixed based on best fit from non-stacked model

'=== BACKGROUND FUNCTION ===
bkg   65.6494664 -56.5905803  88.6485486 -131.799247  146.179609 -130.86077
     90.2523428 -45.8781065  14.8567798  1.99898957 -5.33428946  3.15833661

    PV(,,,, 16282.9057, 11.295, 12.419213, 0.00285109948_LIMIT_MIN_0)

'=== RADIATION SOURCE - Information on the profile of a synchrotron X-ray source ===
Simple_Axial_Model(axial, 1.99023388` ) 'Describes peak asymmetry due to axial divergence
LP_Factor( ,90.0) 'Lorentz-Polarisation factor. Change the monochromator angle as required
lam ymin_on_ymax 0.0001 la 1.0 lo 0.824672 lh 0.1 'Radiation profile

            Specimen_Displacement(,-0.00741947872)

start_X  5
finish_X  80

 '=== Structure with the stacking faults in P1 setting, oriented so stacking occurs along c-axis  ===

                        str
                        phase_name "Y10CuTi4O24"
                        a  = lpc;
                        b  = lpb;
                        c  = Get(generated_c);

'=== Original cell in monoclinic setting, for reference
                        prm  !lpa  12.2457154
                        prm  !lpb  5.86993788
                        prm  !lpc  7.18172143


                        al          90
                        be    72.93256     '           inverted from monoclinicbe @  107.06298`
                        ga          90
                        space_group "P1"

prm !px = 0.041;    'derived optimal value from the grid search
```

```
                    prm s  6.44014647`  min 1e-15
                    scale = s 1e-6/ (Nv Nstr);

                    generate_stack_sequences {
                    pr_str { B layers_tol} 10
                    number_of_sequences Nstr 50
                    number_of_stacks_per_sequence Nv 200      ' increased from 100 due to small value of p
                              Transition(A, lpa)
to A = 1-px;        a_add =  0;  b_add = 0;      'translation in the same cell
to B = px;          a_add =  1/2;  b_add =  0;    'translation in the cell shifted by 1/2 along a in P1 setting
                              Transition(B, A, lpa)
          to A = 1-px;          a_add =  0;  b_add = 0;
          to B = px;            a_add =  1/2;  b_add = 0;

'=== Atomic sites as refined in the monoclinic model===

                    prm    O1x  0.852226549`
                    prm    O1y  0.238680825`
                    prm    O1z  0.0722967165`
                    prm   O1beq  1.36410476`          min 0.1 max 5

                    prm    O2x  0.663113278`
                    prm    O2y  0.268388378`
                    prm    O2z  0.408523653`
                    prm   O2beq  0.773777795`_LIMIT_MIN_0.1          min 0.1 max 5

                    prm    O3x -0.0069468717`
                    prm    O3z  0.316159191`
                    prm   O3beq  0.561006804`_LIMIT_MIN_0.1          min 0.1 max 5

                    prm     Y1x  0.803580302`
                    prm     Y1z  0.345604551`
                    prm    Y1beq  0.753665823`        min 0 max 5

                    prm    O4x  0.492823911`
                    prm    O4z  0.318456274`
                    prm  O4beq  0.100003052`_LIMIT_MIN_0.1          min 0.1 max 5

                    prm     Y2x  0.197343683`
                    prm     Y2z  0.146142609`
                    prm    Y2beq  0.799954977`        min 0 max 5

                    prm     Y3y  0.470426829`
                    prm   Y3beq  0.859819777`         min 0 max 5

                    prm     Ti1y  0.259454975`
                    prm   Ti1beq  0.720961309`        min 0 max 5

                    prm  !Cu1occ  0.5          min 0 max 1
                    prm   Cu1beq  0.957620806`       min 0 max 5
                    volume  98699.720
```

```
'===Restricted atomic sites to preserve initial monoclinic symmetry===

site O1    x  =  (O1z);   y = O1y;       z = (O1x)/Nv;           occ O    1    beq = O1beq;           layer A
site O1002  x  =  (-O1z);  y = O1y;       z = (-O1x)/Nv;           occ O    1    beq = O1beq;       layer A
site O1003  x  =  (-O1z);  y = -O1y;      z = (-O1x)/Nv;           occ O    1    beq = O1beq;       layer A
site O1004  x  =  (O1z);   y = -O1y;      z = (O1x)/Nv;           occ O    1    beq = O1beq;       layer A
site O1005  x  =  (O1z);   y = O1y + 1/2;  z = (O1x + 1/2)/Nv;       occ O    1    beq = O1beq;           layer A
site O1006  x  =  (-O1z);  y = O1y + 1/2;  z = (-O1x + 1/2)/Nv;       occ O    1    beq = O1beq;           layer A
site O1007  x  =  (-O1z);  y = -O1y + 1/2; z = (-O1x + 1/2)/Nv;       occ O    1    beq = O1beq;           layer A
site O1008  x  =  (O1z);   y = -O1y + 1/2; z = (O1x + 1/2)/Nv;       occ O    1    beq = O1beq;           layer A
site O2    x  =  (O2z);    y = O2y;       z = (O2x)/Nv;           occ O    1    beq = O2beq;           layer A
site O2002  x  =  (-O2z);   y = O2y;       z = (-O2x)/Nv;          occ O    1    beq = O2beq;        layer A
site O2003  x  =  (-O2z);   y = -O2y;      z = (-O2x)/Nv;          occ O    1    beq = O2beq;        layer A
site O2004  x  =  (O2z);    y = -O2y;      z = (O2x)/Nv;          occ O    1    beq = O2beq;        layer A
site O2005  x  =  (O2z);    y = O2y + 0.5;  z = (O2x + 0.5)/Nv;      occ O    1    beq = O2beq;            layer A
site O2006  x  =  (-O2z);   y = O2y + 0.5;  z = (-O2x + 0.5)/Nv;      occ O    1    beq = O2beq;            layer A
site O2007  x  =  (-O2z);   y = -O2y + 0.5; z = (-O2x + 0.5)/Nv;      occ O    1    beq = O2beq;            layer A
site O2008  x  =  (O2z);    y = -O2y + 0.5; z = (O2x + 0.5)/Nv;      occ O    1    beq = O2beq;            layer A
site O3    x   =  (O3z);  y 0.00000      z = (O3x)/Nv;            occ O    1    beq = O3beq;       layer A
site O3002  x   =  (-O3z); y 0.00000      z = (-O3x)/Nv;           occ O    1    beq = O3beq;   layer A
site O3003  x   =  (O3z);  y = 1/2;       z = (O3x + 0.5)/Nv;       occ O    1    beq = O3beq;   layer A
site O3004  x   =  (-O3z); y = 1/2;       z = (-O3x + 0.5)/Nv;       occ O    1    beq = O3beq;  layer A
site Y1    x =  (Y1z);    y 0.00000      z = (Y1x)/Nv;          occ Y    1    beq = Y1beq;           layer A
site Y1002  x =  (-Y1z);    y 0.00000      z = (-Y1x)/Nv;         occ Y    1    beq = Y1beq;          layer A
site Y1003  x =  (Y1z);     y 0.50000      z = (Y1x + 0.5)/Nv;      occ Y    1    beq = Y1beq;         layer A
site Y1004  x =  (-Y1z);    y 0.50000      z = (-Y1x + 0.5)/Nv;     occ Y    1    beq = Y1beq;         layer A
site O4    x  =  (O4z);   y 0.00000      z = (O4x)/Nv;            occ O    1          beq = O4beq;    layer A
site O4002  x  =  (-O4z);  y 0.00000      z = (-O4x)/Nv;           occ O    1          beq = O4beq;    layer A
site O4003  x  =  (O4z);   y 0.50000      z = (O4x + 0.5)/Nv;      occ O    1          beq = O4beq;    layer A
site O4004  x  =  (-O4z);  y 0.50000      z = (-O4x + 0.5)/Nv;      occ O    1          beq = O4beq;    layer A
site Y2    x =  (Y2z);     y 0.00000      z = (Y2x)/Nv;         occ Y    1          beq = Y2beq;    layer A
site Y2002  x =  (-Y2z);    y 0.00000      z = (-Y2x)/Nv;        occ Y    1          beq = Y2beq;    layer A
site Y2003  x =  (Y2z);     y 0.50000      z = (Y2x + 0.5)/Nv;    occ Y    1          beq = Y2beq;    layer A
site Y2004  x =  (-Y2z);    y 0.50000      z = (-Y2x + 0.5)/Nv;    occ Y    1          beq = Y2beq;    layer A
site Ti1    x = 0.50000;   y = Ti1y;      z =0.00000/Nv;       occ Ti   1          beq = Ti1beq;    layer A
site Ti1002 x = 0.50000;   y = -Ti1y;      z =0.00000/Nv;       occ Ti   1          beq = Ti1beq;    layer A
site Ti1003 x = 0.50000;   y = -Ti1y +0.5; z =0.50000/Nv;       occ Ti   1          beq = Ti1beq;    layer A
site Ti1004 x = 0.50000;   y = Ti1y + 0.5; z =0.50000/Nv;       occ Ti   1          beq = Ti1beq;    layer A
site Y3    x 0.00000       y = Y3y;      z =0.00000/Nv;   occ Y    0.5                    beq = Y3beq;    layer A
site Y3002  x 0.00000       y = -Y3y;      z =0.00000/Nv;       occ Y    0.5          beq = Y3beq;    layer A
site Y3003  x 0.00000       y = -Y3y +0.5; z =0.50000/Nv;   occ Y    0.5          beq = Y3beq;    layer A
site Y3004  x 0.00000       y = Y3y + 0.5; z =0.50000/Nv;       occ Y    0.5          beq = Y3beq;    layer A
site Cu1    x 0.00000       y 0.00000       z 0.00000        occ Cu   = Cu1occ;           beq = Cu1beq;      layer A
site Cu1002 x 0.00000       y 0.50000       z =0.50000/Nv;      occ Cu   = Cu1occ;        beq = Cu1beq;      layer A

normalize_FCs
                    peak_buffer_based_on = Xo;
                    peak_buffer_based_on_tol 0.1

                    PVII_Peak_Type(@, 0.000100142422`_LIMIT_MIN_0.0001,@, 0.000656316899`,@,
0.0227474472`,@, 0.697453628`,@, 0.000292396457`_LIMIT_MIN_0.0001,@,
0.0001`_LIMIT_MIN_0.0001)
```

```
'=== structural information for inpurities, fixed all except scale and profile according to the best fit

	str
		phase_name "Y2Ti2O7"
		Cubic(   10.099593)
		space_group "Fd-3mz"
		scale @  7.37683024e-07`

		volume  1030.176
		site O1    x  0.42191    y 0.125       z 0.125       occ O   1     beq 0.5
		site Ti1   x 0.5         y 0.5         z 0.5         occ Ti  1     beq  0.80263
		site Y1    x 0           y 0           z 0           occ Y   1     beq  0.99382
		site O2    x 0.125       y 0.125       z 0.125       occ O   1     beq 0.5

		PVII_Peak_Type(@, 0.00111649781`,@, 0.000100045519`_LIMIT_MIN_0.0001,@,
0.017915568`,@, 0.432549399`,@, 0.13528557`_LIMIT_MIN_0.0001,@, 0.0001`_LIMIT_MIN_0.0001)

	str
		phase_name "Y2O3"
		Cubic(   10.602164)
		space_group "206"
		scale @  4.55587853e-07`

		volume  1191.746
		site O1    x  0.09603    y  0.36162    z  0.13151    occ O   1     beq 0.5
		site Y1    x  0.28260    y 0           z 0.25        occ Y   1     beq  0.72048
		site Y2    x 0           y 0           z 0           occ Y   1     beq  0.67215

		PVII_Peak_Type(@, 0.000100599487`_LIMIT_MIN_0.0001,@, 0.00138194047`,@,
0.0341973152`,@, 0.593281014`,@, 0.467825522`,@, 0.0001`_LIMIT_MIN_0.0001)
```

SI5. Additional measurement data of the $Y_{10}Ni_xCu_{1-x}Ti_4O_{24}$ (x = 0, 0.5, 1) phases

Table SI7. Fit parameters using the Curie-Weiss law.

x in $Y_{10}Ni_xCu_{1-x}Ti_4O_{24}$	χ_0 (emu·mol^{-1}·Oe^{-1})	T_{CW} (K)	C (emu·mol^{-1}·Oe^{-1}·K)	μ_{eff} (μ_B)
0	-5.69(6) × 10^{-4}	-10.5(3)	0.577(2)	2.148(3)
0.5	9(2) × 10^{-6}	-5.57(4)	1.1732(7)	3.063(1)
1	3.6(1) × 10^{-5}	-6.65(1)	1.3211(4)	3.251(1)

References:

(1) Barton, P. T.; Premchand, Y. D.; Chater, P. A.; Seshadri, R.; Rosseinsky, M. J. Chemical Inhomogeneity, Short-Range Order, and Magnetism in the $LiNiO_2$-NiO Solid Solution. *Chem. – Eur. J.* **2013**, *19* (43), 14521–14531.

(2) Bharathy, M.; Gemmill, W. R.; Fox, A. H.; Darriet, J.; Smith, M. D.; Hadermann, J.; Remy, M. S.; zur Loye, H. C. Synthesis and Magnetic Properties of Rare Earth Ruthenates, $Ln_5Ru_2O_{12}$ (*Ln*=Pr, Nd, Sm-Tb). *J Solid State Chem* **2009**, *182* (5), 1164–1170.

(3) Plass, M. A.; Bette, S.; Dinnebier, R. E.; Lotsch, B. V. Enhancement of Superionic Conductivity by Halide Substitution in Strongly Stacking Faulted $Li_3HoBr_{6-x}I_x$ Phases. *Chem. Mater.* **2022**, *34* (7), 3227–3235.

(4) Bette, S.; Takayama, T.; Duppel, V.; Poulain, A.; Takagi, H.; Dinnebier, R. E. Crystal Structure and Stacking Faults in the Layered Honeycomb, Delafossite-Type Materials $Ag_3LiIr_2O_6$ and $Ag_3LiRu_2O_6$. *Dalton Trans.* **2019**, *48* (25), 9250–9259.
